# Comprehensive molecular analysis of immortalization hallmarks in thyroid cancer reveals new prognostic markers

**DOI:** 10.1002/ctm2.1001

**Published:** 2022-08-18

**Authors:** Cristina Montero‐Conde, Luis Javier Leandro‐García, Ángel M. Martínez‐Montes, Paula Martínez, Francisco J. Moya, Rocío Letón, Eduardo Gil, Natalia Martínez‐Puente, Sonsoles Guadalix, Maria Currás‐Freixes, Laura García‐Tobar, Carles Zafon, Mireia Jordà, Garcilaso Riesco‐Eizaguirre, Patricia González‐García, María Monteagudo, Rafael Torres‐Pérez, Veronika Mancikova, Sergio Ruiz‐Llorente, Manuel Pérez‐Martínez, Guillermo Pita, Juan Carlos Galofré, Anna Gonzalez‐Neira, Alberto Cascón, Cristina Rodríguez‐Antona, Diego Megías, María A. Blasco, Eduardo Caleiras, Sandra Rodríguez‐Perales, Mercedes Robledo

**Affiliations:** ^1^ Hereditary Endocrine Cancer Group Human Cancer Genetics Program Spanish National Cancer Research Centre (CNIO) Madrid Spain; ^2^ Biomedical Research Networking Centre on Rare Diseases (CIBERER) Institute of Health Carlos III Madrid Spain; ^3^ Telomeres and Telomerase Group Molecular Oncology Program Spanish National Cancer Research Centre (CNIO) Madrid Spain; ^4^ Molecular Cytogenetics Unit Human Cancer Genetics Program Spanish National Cancer Research Center (CNIO) Madrid Spain; ^5^ Department of Endocrinology Hospital Universitario 12 de Octubre Madrid Spain; ^6^ Department of Endocrinology Clínica Universidad de Navarra Madrid Spain; ^7^ Familial Cancer Clinical Unit Human Cancer Genetics Program Spanish National Cancer Research Centre (CNIO) Madrid Spain; ^8^ Anatomical Pathology Section Clínica Universidad de Navarra Pamplona Spain; ^9^ Department of Endocrinology Hospital Universitari Vall d'Hebron Barcelona Spain; ^10^ Program for Predictive and Personalized Medicine of Cancer (PMPPC) Germans Trias i Pujol Research Institute (IGTP) Badalona Spain; ^11^ Department of Endocrinology and Nutrition Hospital Universitario de Móstoles Madrid Spain; ^12^ Endocrinology Molecular Group, Faculty of Medicine Universidad Francisco de Vitoria Madrid Spain; ^13^ Histopathology Unit Biotechnology Program, Spanish National Cancer Research Center (CNIO) Madrid Spain; ^14^ Bioinformatics for Genomics and Proteomics National Centre for Biotechnology (CNB‐CSIC) Madrid Spain; ^15^ Department of Biomedicine and Biotechnology Universidad de Alcalá (UAH) Alcalá de Henares Spain; ^16^ Confocal Microscopy Unit Biotechnology Program, Spanish National Cancer Research Center (CNIO) Madrid Spain; ^17^ CEGEN Unit Human Cancer Genetics Program Spanish National Cancer Research Centre (CNIO) Madrid Spain; ^18^ Department of Endocrinology Clínica Universidad de Navarra Pamplona Spain; ^19^ Instituto de Investigación Sanitaria de Navarra (IdiSNA) Pamplona Spain

**Keywords:** 5p‐end FISH, subtelomeric gene expression, telomere shortening, TERC, TERT promoter methylation, TERT promoter mutation

## Abstract

**Background:**

Comprehensive molecular studies on tumours are needed to delineate immortalization process steps and identify sensitive prognostic biomarkers in thyroid cancer.

**Methods and Results:**

In this study, we extensively characterize telomere‐related alterations in a series of 106 thyroid tumours with heterogeneous clinical outcomes. Using a custom‐designed RNA‐seq panel, we identified five telomerase holoenzyme‐complex genes upregulated in clinically aggressive tumours compared to tumours from long‐term disease‐free patients, being *TERT* and *TERC* denoted as independent prognostic markers by multivariate regression model analysis. Characterization of alterations related to *TERT* re‐expression revealed that promoter mutations, methylation and/or copy gains exclusively co‐occurred in clinically aggressive tumours. Quantitative‐FISH (fluorescence in situ hybridization) analysis of telomere lengths showed a significant shortening in these carcinomas, which matched with a high proliferative rate measured by Ki‐67 immunohistochemistry. RNA‐seq data analysis indicated that short‐telomere tumours exhibit an increased transcriptional activity in the 5‐Mb‐subtelomeric regions, site of several telomerase‐complex genes. Gene upregulation enrichment was significant for specific chromosome‐ends such as the 5p, where *TERT* is located. Co‐FISH analysis of 5p‐end and *TERT loci* showed a more relaxed chromatin configuration in short telomere‐length tumours compared to normal telomere‐length tumours.

**Conclusions:**

Overall, our findings support that telomere shortening leads to a 5p subtelomeric region reorganization, facilitating the transcription and accumulation of alterations at *TERT‐locus*.

## Graphical abstract

This article comprehensively characterizes molecular alterations associated with immortalization and telomere length of a thyroid tumor series comprising the different clinical behaviors of the disease. The study proposes a model for the immortalization process and unveils a new marker of disease prognosis based on the chromatin spatial organization of 5pter and *TERT*‐locus.

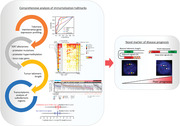

## 1 BACKGROUND

Cell immortalization is a hallmark in tumour progression that requires the activation of a mechanism to overcome the replicative barrier established by telomere shortening with each cell division. Telomeres are repetitive sequences of the nucleotide hexamer TTAGGG located at chromosomal ends, which, together with the shelterin protein complex, protect chromosomes from end‐to‐end fusions and degradation.^1^

Telomerase complex is responsible of telomere length maintenance. It consists of a catalytic subunit, the telomerase reverse transcriptase gene, *TERT* and an intrinsic template of RNA, *TERC*, as well as accessory proteins such as *DKC1*, *NOP10*, *NHP2*, *GAR1* and *TCAB1* among others, all necessary for the tuned activity of telomerase.^2^ Telomerase activation is the most frequent mechanism of immortalization in cancer.^3^ It requires the re‐expression of *TERT*, which is silenced in somatic cells.^4^ The re‐expression of *TERT* in cancer cells allows the repair of critically short telomeric lengths and maintenance of an unlimited replication rate in cancer cells.

First identified pathogenic alterations associated with *TERT* re‐expression in cancer were the c.‐124C>T (C228T) and c.‐146C>T (C250T) *TERT* promoter mutations.^5^, ^6^ Since then, a plethora of mechanisms associated with its pathogenic expression have been identified, including promoter methylation,^7^ focal copy number gains,^8^ alternative splicing^9^, ^10^ and super‐enhancer rearrangements.^11^

Particularly in thyroid cancer, mutations in the *TERT* promoter are present in 73% of advanced disease histologies, the dedifferentiated carcinomas.^12^ In differentiated forms, *TERT* promoter mutations are found in ∼9% of the tumours and are associated with disease outcome.^13^, ^14^, ^15^, ^16^ Similarly, studies aimed at the analysis of *TERT* promoter methylation also point to methylation as a recurrent event in clinically aggressive thyroid tumours.^17^, ^18^ Altogether, these findings suggest that alterations related to *TERT* re‐expression are late events in thyroid tumourigenesis. However, the prognostic specificity of *TERT* promoter mutations and expression has been questioned as they have also been reported in benign forms of thyroid^19^, ^20^ and other types of cancer,^21^ highlighting the need of further studies to refine the prognostic value of these immortalization markers.

Analysis of tumour telomere length has recently shown to be critical to understand the kinetics of the pathogenic events related to immortalization in each cancer type.^8^ Notably, telomere shortening in untransformed yeast and mammals cells leads to structural changes at the end of chromosomes^22^, ^23^ that can affect several megabases (Mb), delineating gene regulation at subtelomeric sites.^24^ In humans, *TERT locus* is located only 1.2 Mb from the end of chromosome 5p, and experiments in cellular models have demonstrated that *TERT* is sensitive to the regulatory mechanism named TPE‐OLD (telomere position effect over long distances),^25^ which could be relevant in the context of cancer.^8^

To establish a framework to develop new prognostic tools in thyroid cancer, we comprehensively characterize molecular alterations associated with immortalization and telomere length of a tumour series that comprise the different clinical behaviours of the disease. Transcriptional analysis of telomere maintenance genes reveals an upregulation of *TERT* and other four telomerase holoenzyme complex genes in poor prognosis tumours. Integration analysis of telomere lengths with *TERT* promoter alterations and subtelomeric gene expression patterns prompted us to explore the chromatin organization of 5p subtelomeric region in tumours, leading to propose a new prognostic marker for thyroid cancer.

## 2 MATERIALS AND METHODS

### 2.1 Thyroid tumour tissues and patients

A total of 76 fresh‐frozen and 30 FFPE thyroid tumour specimens from 96 patients, including 3 with benign tumour disease (follicular adenomas), 81 with differentiated thyroid cancer (DTC) and 12 with anaplastic thyroid cancer (ATC), were analysed by a custom RNA expression panel. Frozen and FFPE specimens were first evaluated by a pathologist (E. Caleiras). All tumours included in the study held a tumour content superior to 80%.

Disease‐free primary tumours were defined as tumours from patients with excellent response to initial therapy (structural and biochemical complete response), no evidence of disease, recurrence or disease specific mortality along the follow‐up (median = 100 months; range = 16–180 months), this being superior to 60 months for all but four patients. Clinically aggressive tumours were those from patients who did not fit one or more of these conditions, showing persistent or progressive disease at the latest available follow‐up date and/or with an advanced histology tumour (ATC or poorly DTC) (Table 1). Associated clinicopathological data are summarized in Table 1 and detailed in Table S1.^26^ Specific clinicopathological characteristics of DTC patients dichotomized into disease‐free and clinically aggressive disease are summarized in Table S2. Series was enriched with clinically aggressive tumours.

**TABLE 1:** Clinicopthological features of the patients and primary tumours according to the AJCC/TNM staging system (eighth Edition) for differentiated (DTC) and anaplastic (ATC) thyroid cancer.

	All patients (*n* = 93)	DTC patients (*n* = 81)	ATC patients (*n* = 12)
*Variables* (categories)	*n* (%)	*n* (%)	*n* (%)
*Age*			
<55	53 (57.0)	53 (65.4)	0 (.0)
≥55	40 (43.0)	28 (34.6)	12 (100.0)
*Sex*			
Female	66 (71.0)	58 (71.6)	8 (66.7)
Male	26 (28.0)	22 (27.2)	4 (33.3)
Not available	1 (1.1)	1 (1.2)	0 (.0)
*Primary tumour (T)*			
T1	21 (22.6)	21 (25.9)	0 (.0)
T2	27 (29)	25 (30.9)	2 (16.7)
T3	33 (35.5)	31 (38.3)	2 (16.7)
T4	8 (8.6)	2 (2.5)	6 (50.0)
T*X*	4 (4.3)	2 (2.5)	2 (16.7)
*Regional lymph node metastases at dx (N)*			
N0	26 (28.0)	24 (29.6)	2 (16.7)
N1	42 (45.2)	34 (42.0)	8 (66.7)
N*X*	25 (26.9)	23 (28.4)	2 (16.7)
*Distant metastases at dx (M)*			
M0	61 (65.6)	60 (74.1)	1 (8.3)
M1	28 (30.1)	19 (23.5)	9 (75.0)
M*X*	4 (4.3)	2 (2.5)	2 (16.7)
*AJCC (prognostic groups)^a^*			
I	51 (54.8)	51 (63.0)	0 (.0)
II	17 (18.3)	17 (21.0)	0 (.0)
III	1 (1.1)	1 (1.2)	0 (.0)
IV	22 (23.7)	10 (12.3)	12 (100.0)
Not available	2 (2.2)	2 (2.5)	0 (.0)
*Disease outcome*			
Excellent response after initial treatment^b^	55 (59.1)	55 (67.9)	0 (.0)
Disease progression	24 (25.8)	20 (24.7)	4 (33.3)
Biochemical evidences of disease	1 (1.1)	1 (1.2)	0 (.0)
Locoregional lymph node recurrence^c^	9 (9.7)	8 (9.9)	1 (8.3)
Distant metastasis in progression	1 (1.1)	1 (1.2)	0 (.0)
Exitus due to the disease	13 (14.0)	10 (12.3)	3 (25.0)
Persistent disease (M1 at *dx*)	4 (4.3)	4 (4.9)	0 (.0)
Follow‐up data not available^d^	10 (10.8)	2 (2.5)	8 (66.7)

^a^ ATC are all AJCC stage IV.

^b^Follow‐up of the 92.7% (51/55) is ≥60 months.

^c^Only second surgeries material is available for four of these patients.

^d^Eight ATC and two DTC with poorly differentiated component.

### 2.2 Genetic material extraction and integrity analysis

Total RNA and genomic DNA of fresh frozen samples were isolated using a TRIzol reagent (Sigma Aldrich, T9424) and a FlexiGene DNA kit (Qiagen, Cat No. 51206), respectively, according to the manufacturer's protocol. Two 10‐μm sections of FFPE specimens were used for total RNA and DNA extraction using either the truXTRAC FFPE RNA kit (Covaris, cat no 520161) or the truXTRAC FFPE DNA microTUBE kit (Covaris, Cat No. 520136). DNA was quantified by Quant‐iT PicoGreen (Thermo Fisher Scientific). RNA concentration was determined by NanoDrop 1000 (Thermo Fisher Scientific), and integrity was assessed using an Agilent Bioanalyzer 2100 (Agilent Technologies). The percentage of RNA fragments superior to 200 nt (DV_200_) was determined to identify those RNAs with an extreme poor integrity (DV_200_ < 30%).

### 2.3 Custom RNA sequencing panel and data analysis

We designed a TruSeq Targeted RNA expression (TREx, Illumina) panel that assesses the expression of 187 genes represented by 227 probes,^27^ including 26 genes involved in telomere maintenance. These telomere maintenance genes encode main components of the telomerase holoenzyme: TERT, TERC; telomerase accessory proteins: DKC1, GAR1, NOP10, NHP2, NAT10, TCAB1 (WRAP53), TEP1; shelterin–telosome complex proteins: POT1, TERF1, TERF2, TERF2IP, TINF2, ACD (TPP1); components of the alternative telomere lengthening mechanism: ATRX, DAXX; and proteins indirectly involved in telomere maintenance: MKRN1, NFX1, YLPM1, MCRS1, TNKS, FBXO4, RLIM, SMG5, SMG6. Four *TERT* probes were included in the assay to increase the sensitivity of detection of *TERT* expression. The identifiers of the probes and the transcripts quantified by these probes are detailed in Supporting Information Methods Table S1.^26^

RNAs from fresh‐frozen specimens and formalin‐fixed tissues that passed the previously indicated RNA integrity criteria (DV_200_ ≥ 30%) were used for library generation of custom RNA expression panel following Illumina's protocol (Part 15034665). RNA input was adjusted to DV_200_ values according to manufacturer's recommendations (DV_200_ > 70%, 200 ng; DV_200_ = 50%–70%, 400 ng; DV_200_ = 30%–50%, 600 ng). High‐quality commercial RNA from 10 human cell lines (Universal Human Reference RNA, Agilent Technologies, #740000) and RNA from a thyroid tumour was included in all runs as inter‐assay control. Libraries were prepared for sequencing in an Illumina MiSeq system following Illumina's instructions (Part 15039740) and three sequencing runs of 50‐bp single reads were approached. The output datasets were mapped to the reference genome version GRCh37, adapted for the TREx custom panel, using TopHat^28^ included in the Nextpresso suite.^29^ Total mapped reads were considered to evaluate the sequencing and mapping steps and to perform a random down‐sampling that enable the comparison within samples. Samples with less than 170 000 aligned reads were discarded due to low read depth (<700 reads/amplicon). A total of 112 samples (36 FFPE and 76 Frozen) passed the cut‐off and their FASTQ files were randomly down‐sampled to 170 000 reads. New mapping process was performed with the down‐sampled files and different quality steps were assessed. First, we tried to remove the bias effect generated by differences between FFPE and frozen samples, using limma package^30^ to process the data, obtaining unbiased log2CPMs (log_2_ counts per million) for all the selected samples. Outliers identified when evaluating the number of genes with extreme expression values for each sample in the whole series were discarded, 6 FFPE samples with >125 genes in the 10% of the higher or lower extreme values of the series were excluded.

Specifically for *TERT* expression, all samples with less than three normalized reads for the four *TERT* probes included in the assay were considered *TERT* expression negative. Telomere maintenance genes with different expressions between disease free and clinically aggressive tumours were defined as those genes with a two‐tailed Mann–Whitney *U*‐test *p*‐value <.05 (False Discovery Rate (FDR)‐adjusted *p*‐value <.15). Genes with a twofold higher frequency of extreme expression outliers in clinically aggressive tumours were also considered candidates genes for further analyses. Extreme gene expression outliers were defined as those with expression values below or above lower/upper whiskers (*Q*1 − (1.5 × IQR [interquartile range]) or *Q*3 + (1.5 × IQR), respectively) of the gene expression dispersion disease‐free tumours (reference tumour set). Supporting Information Methods Table S1, ^26^ includes the normalized gene expression matrix of telomere maintenance genes for the 106 analysed tumour samples and the unadjusted and FDR‐adjusted *p*‐values of two‐tailed Mann–Whitney *U*‐tests performed.

### 2.4 Identification of hotspot mutations in *TERT* promoter

The PCR amplicon was performed following the protocol ‘16S Metagenomic Sequencing Library Preparation’ (Illumina). First, the regions with hotspot mutations in the *TERT* promoter was amplified by PCR. The sequences of the primers used are listed in Supporting Information Methods Table S2. Amplicon PCR was performed using the DNA polymerase enzyme from the Multiplex kit (QIAGEN) following the manufacturer's instructions. PCR products were purified using AMPure XP beads (Beckman Coulter). Index PCR was later performed with the EasyTaq DNA polymerase (TransGen Biotech) using synthetic indexes from the Nextera XT Index kit (Illumina). Index PCR products were purified with AMPure XP beads and quantified by PicoGreen (Thermo Fisher Scientific). The concentration of each amplicon was normalized, and library pooling was performed by mixing equal volumes of each amplicon with unique indexes.

Libraries were sequenced on an MiSeq sequencer (Illumina), following the protocol provided by the manufacturer. The sequencing module used was the ‘PCR Amplicon’ protocol with a paired‐end design with 150 base pairs reads of length. The reference genome used for reads alignment was GRCh37 and Illumina Variant Studio software v3.0 was used for the annotation of the mutations.

### 2.5 Analysis of *TERT* promoter methylation levels

To analyse *TERT* promoter methylation *status*, bisulfite‐modified DNA was used as described in Lee et al.^17^ A *TERT* promoter region comprising 39 CpG sites within *TERT* hypermethylated oncological region (THOR) (*n* = 29) and the proximal region to *TERT* transcription start site (*n* = 10) was divided in four PCR amplicons. Primers’ sequences are listed in Supporting Information Methods Table S2. Within the amplicon THOR_A4 is located the UTSS region (upstream of the transcription start site) containing a subset of five CpG sites (CpG 1295586, 1295590, 1295593, 1295605 and 1295618) whose average methylation level accurately correlates with the average methylation level of the whole analysed region.^17^ An amount of 100‐ng genomic DNA was bisulfite‐modified with the EZ‐96 DNA MethylationTM kit (ZYMO RESEARCH), following the protocol provided by the manufacturer. Preparation and sequencing of the four amplicons were performed following the protocol ‘16S Metagenomic Sequencing Library Preparation’ for the Illumina MiSeq system as previously described for the hotspot mutations in a *TERT* promoter.

The FASTQ files of each samples were generated, and forward reads were trimmed with cutadapt software v1.18 to eliminate the sequences corresponding to the Illumina sequencing adapters. Analysis was performed with BS‐Seeker2 software v2.1.8,^31^ used to measure methylation levels after bisulfite modification. The first step was the generation of a reference genome adapted to bisulfite modification based on human reference genome GRCh37. Reads were aligned to this modified genome using BS‐Seeker2 v2.1.8.^31^ The result of this process is a new alignment (BAM file), which incorporates information on methylation levels. Two steps were carried out to interpret these data. The first was to obtain the coverage at the positions of interest using a script developed in the laboratory based on bam‐read count software (0.8.0‐unstable‐6‐963acab [commit 963acab]) (https://github.com/genome/bam‐readcount). The second step was carried out based on previous results, which were used to calculate the percentage of methylation observed for each CpG site as a function of the number of reads reporting a ‘C’ divided by the total number of reads reporting a C or T at that position. Mean methylation percentages of all analysed CpG sites (*n* = 29) and UTSS‐specific CpG sites (*n* = 5) were calculated. The cut‐off value of 16.1% was used to dichotomize tumours in *TERT* promoter methylated (≥16.1%) or non‐methylated (<16.1%). This threshold was established by Lee et al.,^17^ which corresponds to THOR methylation mean value plus 2SDs of a set of 80 normal tissues from different cell lineages.

### 2.6 SNP array and calculation of proportion of cells harbouring somatic deletions and amplifications

Genomic DNA from 31 thyroid tumours (Table S1; column: ‘*TERT locus* copy gain’^26^) were analysed with the Illumina Infinium Global Screening Array (including 660 000 SNPs) following the manufacturer's recommendations. The assay was run on the Illumina BeadStation by the Human Genotyping‐CEGEN Unit at the CNIO. Cancer cell component of each tumour was calculated using an ASCAT bioinformatics tool, which estimates the aberrant cell fraction of solid tumours considering ploidy and allele‐specific copy number.^32^ Briefly, this algorithm uses B‐allele‐frequency and Log*R* tracks obtained from GenomeStudio v2.0 software and preprocesses them by the specifically designed segmentation and filtering algorithm, Allele‐Specific Piecewise Constant Fitting, to cluster probes into segments. As only tumour tissue was available for analysed samples, we inferred germline genotypes from the tumour data. ASCAT R package is available at https://github.com/Crick‐CancerGenomics/ascat. Focal copy number for autosomal chromosomes was estimated with a GISTIC2 tool,^33^ using a segmentation file for each tumour sample obtained with DNAcopy R package.

### 2.7 Telomere length Q‐FISH and high‐throughput quantification

Telomere length was studied by Q‐FISH (quantitative fluorescence in situ hybridization) in OCT‐embedded frozen specimens, including 55 thyroid tumours of the series and 9 normal thyroid tissues. Normal thyroid tissues were used to establish the reference of normal telomeric length. Normal thyroid tissues corresponded to six individuals without thyroid disease who went through a laryngectomy and three thyroid cancer patients included in the study. The ages ranged from 50 to 82 years (median = 67‐year old).

Briefly, 3‐μm‐thick complete sections of OCT embedded frozen tissues were mounted on Thermo Scientific SuperFrost plus slides and preserved at −20°C until experiment performance. First, slides were defrosted at room temperature for 5 min. Tissues were washed in PBS 1× and fixed in 4% formaldehyde for 5 min. After washing, slides were dehydrated in a 70%–90%–100% ethanol series (5 min each). Slides were air dried and 30 μl of the telomere probe mix (10‐mM Tris–HCl, pH 7.2, 25‐mM MgCl_2_, 9‐mM citric acid, 82‐mM Na_2_HPO_4_, 50% deionized formamide (Sigma‐Aldrich, Darmstadt, Germany), .25% blocking reagent (Roche, Basel, Switzerland) and .5‐μg/ml Telomeric PNA probe (Panagene, Daejeon, Korea) was added. Slides were incubated for 3 min at 85°C and then 2 h at room temperature in a wet chamber in the dark. Slides were washed twice for 15 min each in 10‐mM Tris–Cl (pH 7.2) and .1% BSA in 50% formamide and then three times for 5 min each in TBS .08% Tween 20. After washing, slides were stained with DAPI (4',6‐diamidino‐2‐phenylindole; .2 μg/ml) and dehydrated in a 70%–90%–100% ethanol series. Dried samples were finally mounted with VECTASHIELD mounting media (Vector Laboratories, Burlingame, CA, USA). Telomere length analysis is based on the specific and stable hybridization of the PNA with the telomeric region; the intensity of this PNA is directly related to telomere length allowing the measurement of telomeres at each individual chromosome end. Samples were imaged and quantified by confocal microscopy. For each sample evaluation, five representative areas from each tumour were imaged for an unbiased study of telomere length. Q‐FISH images were acquired in a confocal microscope equipped with a 63×/NA 1.4 oil immersion objective and LAS AF v2.6 software (Leica Microsystems, Wetzlar, Germany), and maximum projection images were created with the LAS AF 2.7.3.9723 software. Telomere signal intensity from Z‐stacks was quantified using Definiens Developer Cell software version XD 64 2.5. Telomere length was estimated as the mean telomere intensity value *per nucleus*.

### 2.8 Immunohistochemical assays and evaluation

Complete sections of OCT‐embedded frozen tissues with a thickness of 3 μm were mounted on Thermo Scientific SuperFrost plus slides and fixed in acetone for 10 min. Slides were rehydrated through a series of graded ethanol solutions and finally rinsed in water. Immunohistochemistry reactions were performed in an automated immunostaining platform (Autostainer Link 48, Dako) following standard instructions. Endogenous peroxidase was blocked with 3% hydrogen peroxide solution in methanol for 15 min. Next, slides were incubated with either FLEX Mouse Monoclonal anti‐human Ki‐67 (clone MIB‐1, pre‐diluted, DAKO, IR626) or FLEX mouse monoclonal anti‐human CD45 leukocyte common antigen (LCA) (clones 2B11+PD7‐26, pre‐diluted, DAKO, IR751). After the primary antibody, slides were incubated with the visualization systems (EnVision FLEX, Dako) conjugated with horseradish peroxidase. Slides were developed with 3,3′‐diaminobenzidine tetrahydrochloride (DAB) (DAB+, Dako) and nuclei were counterstained with Carazzi's hematoxylin. The slides were dehydrated, cleared and mounted with a permanent mounting medium for microscopic evaluation. Immunochemistry protocol was carried out by the CNIO Histopathology Core Unit.

Ki‐67 and CD45 slides were scanned with the AxioScan.Z1 slide scanner system (Zeiss, Jena, Germany). Ki‐67 and CD45 immunostaining images were quantified with the ZEISS ZEN 3.1 (blue edition) automatic image analysis software. An expert pathologist (E. Caleiras), who was blinded to the clinical and molecular data, validated the automatic quantification of Ki‐67 and CD45 immunostaining.

### 2.9 RNA sequencing and data analysis

An amount of 1‐μg total RNA from 15 frozen tumour specimens of the initial series (10 short telomere‐tumours and 5 normal telomere tumours) were used to prepare RNA‐seq libraries by the CNIO Genomics Unit. PolyA+ fraction was purified and randomly fragmented, converted to double‐stranded cDNA and processed through subsequent enzymatic treatments of end‐repair, dA‐tailing and ligation to adapters with the ‘NEBNext Ultra II Directional RNA Library Prep Kit for Illumina’ (NEB, Cat. No. E7760) as recommended by the manufacturer. This kit incorporates dUTP during second strand cDNA synthesis, which implies that only the cDNA strand generated during first strand synthesis is eventually sequenced. The adapter‐ligated library was completed by PCR with Illumina PE primers. The resulting purified cDNA library was applied to an Illumina flow cell for cluster generation, and the generated libraries were sequenced for 50 bases in a single‐read format on an Illumina HiSeq 2000 sequencer following the manufacturer's protocols. Image analysis, per‐cycle base calling and quality score assignment were performed with Illumina Real Time Analysis software (RTA) v1.17.21.3. Conversion of BCL files to FASTQ format was performed with the bcl2fastq software v2.20 (Illumina). Overall, 51‐bp single‐reads were analysed with the Nextpresso pipeline^29^ as follows: Sequencing quality was checked with FastQC v0.11.0 (http://www.bioinformatics.babraham.ac.uk/projects/fastqc/), reads were aligned to the human genome (GRCh37/hg19) with TopHat‐2.0.10^28^ and Bowtie 1.0.0,^34^ allowing 2 mismatches and 20 multihits. Expression count normalization was performed using DESeq2.^35^ Differential expression test was performed using moderated limma *t*‐test with the Pomelo II web‐based tool.^36^

### 2.10 Fluorescence in situ hybridization (FISH) of chromosome 5p end and *TERT locus*

Two bacterial artificial chromosome (BAC) clones, RP11‐990A6 for hTERT locus staining and RP11‐44H14 for sub‐telomeric region 5p staining were purchased from BACPAC Resources Center (https://bacpacresources.org/) to generate directly labelled FISH probes. Probes were prepared using nick translation kits (Abbott Laboratories, Abbott Park, IL, USA) from each BAC following manufacturer's instructions. RP11‐990A6 was labelled in Spectrum Orange and RP11‐44H14 in Spectrum Green. All samples were sectioned at 4–6 μm using a refrigerated microtome and mounted on positively charged slides (Thermo Scientific). FISH analyses were performed using the Histology FISH Accessory Kit (DAKO) following the manufacturer's instructions.^37^ Briefly, the slides were pre‐treated in 2‐[*N*‐morpholino]ethanesulfonic acid, followed by a 30‐min protein digestion performed in pepsin solution. After dehydration, the samples were denatured in the presence of the FISH probes at 73°C for 5 min and left overnight for hybridization at 37°C. Finally, the slides were washed with 20× SSC (saline‐sodium citrate) buffer with detergent Tween‐20 at 63°C and mounted with fluorescence mounting medium (DAPI in antifade solution). The proximity of allele pairs was determined visually by a fluorescence microscope (Leica Microsystems CMS GmbH, DM5500B) and quantitated. At least 200 nuclei were counted for the statistical analyses and signal pairs classified as adjacent or separated according to signals’ proximity as indicated in Figure 5A. FISH images were also captured using a CCD camera (CV‐M4+CL Mega Pixel Progressive Scan camera) connected to a PC running the CytoVision v 7.4.0.0 image analysis system (Applied Imaging Ltd., UK) with focus motor and Z‐stack software. FISH analysis was performed by a qualified cytogenetic technologist (F. J. Moya) and interpreted by a cytogeneticist (S. Rodríguez‐Perales).

To validate fluorescence microscope quantification, a set of slides (*n* = 6) was captured in a TCS‐SP8 (AOBS) confocal microscope (Leica microsystems) by using a 100× HC PL APO CS2 oil immersion objective at CNIO Confocal Microscopy Core Unit. Z‐stack images were acquired (each .45 μm) with a pixel size of .114 μm to calculate the distance (μm) between green and red signals. Signal pairs were defined as those with a maximum distance of .7 μm between the limit of the red signal and the proximal green signal. Image segmentation was performed by using a custom made rule set programmed in Definiens developer XD v2.5 software.

### 2.11 Statistics

**FIGURE 1:**
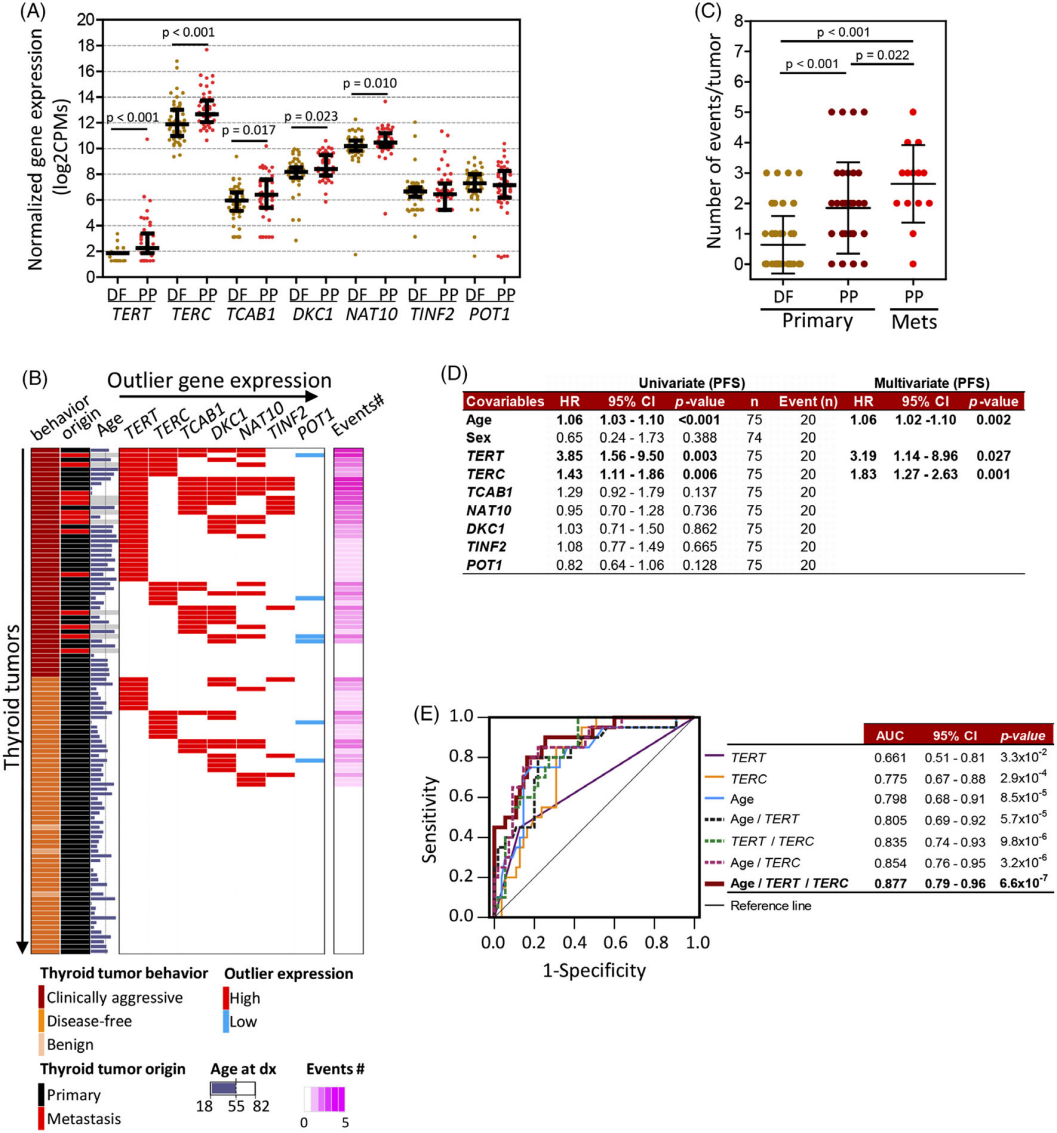
Clinically aggressive thyroid tumours accumulate gene expression outliers among telomerase complex related genes. (A) Gene expression plot showing differentially expressed genes between tumours from disease‐free patients (DF, *n* = 58) and clinically aggressive tumours (poor prognosis [PP], *n* = 48). DF tumours are coloured in orange and PP tumours in red. Black bars represent expression median and interquartile ranges (IQRs) of each represented gene for DF and PP tumour sets. Genes with a two‐tailed Mann–Whitney test *p*‐value <.05 (FDR‐adjusted *p*‐value <.15) or genes with a twofold increase in the frequency of the number of extreme expression outliers (*Q*1 − (IQR × 1.5) or *Q*3 + (IQR × 1.5)) in PP tumours compared to DF tumours are represented. Median, IQR, *Q*1 and *Q*3 refer to the median, IQR, quartile 1 and quartile 3, respectively, of gene expression values of the DF tumour set. (B) Outlier gene expression print of indicated genes for PP tumours (*n* = 48) and DF tumours (*n* = 58). Bright red highlights gene expression values higher than (median + IQR) for *TERT*, *TERC*, *TCAB1*, *DKC1* and *NAT10* genes or *Q*3 + (IQR × 1.5) value for *TINF2*, and bright blue highlights values lower than *Q*1 − (IQR × 1.5) value for *POT1*. Median, IQR, *Q*1 and *Q*3 refer to the median, IQR, quartile 1 and quartile 3, respectively, of gene expression values of the DF tumour set. Event number represents the number of genes with outlier expression for each tumour. Age stands for age at diagnosis (*dx*). (C) Plot representing the number of gene expression outliers for the identified 7‐gene set stratified according to tumour origin (primary or metastasis) and clinical behaviour of the disease. Black bars represent a mean number of outliers and SD for each tumour set. One‐tailed Mann–Whitney test *p*‐values are shown, ***p ≤ .001 and *p *≤* .05. (D) Univariate and multivariate Cox regression model results to evaluate age, sex and the expression of indicated genes as risk factors for hazard of progressive disease (*n* = number of patients; Event (*n*) = number of patients with event). All variables were continuous except sex (male/female) and *TERT* expression (positive/negative). Forward stepwise (likelihood ratio) regression method was used for multivariate modelling on progression‐free survival (PFS). HR, hazard ratio; 95%CI, confidence intervals. (E) Receiver‐operating characteristic (ROC) curve analysis of indicated variables and their combination for the prediction of progressive disease. AUC, area under the curve; 95%CI, confidence intervals

General statistical analyses were conducted using GraphPad Prism v5.03 and SPSS v19.0 (IBM SPSS Statistics, Armonk, NY). Fisher's exact test was used to analyse the association between categorical variables and two‐tailed Mann–Whitney *U*‐test to analyse the association between continuous variables. For directional hypothesis, such as that clinically aggressive tumours accumulate more gene expression outliers than tumours from disease‐free patients, one‐tailed Mann–Whitney *U*‐test was assessed to compare the medians between tumour groups. Progression‐free survival was defined as the time between the date of primary surgery and either the date of local recurrence or distant metastasis, or death from thyroid cancer disease. The Kaplan–Meier method was used to calculate time to progression, and the log‐rank test was used to determine whether differences in the outcomes were statistically significant. Univariate Cox regression or Cox proportional hazard regression analysis was used to analyse the effect of the expression of candidate genes and clinicopathological variables: age at diagnosis, sex and AJCC‐stage on progression‐free survival as single variables. Only variables with significant *p*‐values (*p*‐value <.05) were included in the forward stepwise Cox regression modelling analysis to identify possible predictors of progression‐free survival. Variables included in the final step of the procedure are shown in the multivariate Cox regression tables (Figure 1D and Figure S2B) and represent independent risk factors of disease progression to be explored in further studies. Receiver operating characteristic (ROC) curve analysis for prediction of disease progression was assessed for these variables and their combinations to determine their discriminatory power (area under the curve, AUC). Association analysis of tumour median telomere length with categorical variables (disease clinical behaviour, Figure 3A, and *status* for *TERT* promoter mutation, Figure 3C) were performed by binary logistic regression analysis and adjusted for the covariate age at diagnosis.

Correlation between continuous variables was estimated by Pearson's correlation coefficient (*r*). Specific statistical analyses are detailed in Section 3 and figure legends. *p‐*Values *≤*.05 were considered as statistically significant.

### 2.12 Study approval

Tumours and normal thyroid tissues were collected from Hospital Universitario 12 de Octubre, Hospital Vall d'Hebron, Hospital Universitari Germans Trias i Pujol with the approval of their respective institutional ethics committee (approval number 15/024, PR(AG)90/2015 and PI‐15‐018, respectively), as well as, the CNIO Tumor Bank. Patients completed a written informed consent prior to inclusion in the study.

### 2.13 Data availability

The human samples mRNA sequencing data used for the analysis of the expression of subtelomeric genes compared to non‐subtelomeric genes (*n* = 15) are deposited in the Gene Expression Omnibus (GEO) at www.ncbi.nlm.gov/geo/, reference number GSE193623. Table S1 and Supporting Information Methods Table S1 are openly available in Figshare repository, at https://doi.org/10.6084/m9.figshare.19704208 ^26^. The remaining data and methods are available within the article and the Supporting Information section or from the corresponding authors upon reasonable request.

## 3 RESULTS

### 3.1 Clinically aggressive thyroid tumours exhibit an upregulation of telomerase complex genes

To capture a comprehensive snapshot of immortalization hallmarks in thyroid cancer, we profile gene expression patterns of 26 telomere maintenance–related genes through an RNA‐seq custom panel in a series of 106 thyroid tumour specimens (Table S1 and Supporting Information Methods Table S1, ^26^) corresponding to 96 patients (Table 1 and Table S2). Five genes encoding telomerase holoenzyme complex main components: *TERT*, *TERC*, and accessory proteins: *DKC1*, *TCAB1* and *NAT10*, showed significant differential expression between primary thyroid tumours with excellent response after initial treatment (*n* = 58) and clinically aggressive tumours (*n* = 48) (34 primary and 14 metastases) (two‐tailed Mann–Whitney *U*‐test *p*‐value < .05) (Figure 1A). In addition, the shelterin‐complex genes: *TINF2* and *POT1* exhibited a twofold increased number of expression outliers among clinically aggressive tumours (Figure 1A). Notably, outlier expressors of the seven identified candidate genes significantly co‐occur among clinically‐aggressive tumours (Figure 1B,C), also denoted by the progressive accumulation of expression outliers in disease‐free associated tumours to clinically aggressive primary tumours (*p* ≤ .001) and from the latest to metastases (*p* = .022) (Figure 1C). Similarly, the analysis of identified candidate genes in the TCGA thyroid cancer series showed that the accumulation of telomerase holoenzyme complex outlier gene expressors was significantly enriched for tumours with the highest values of MACIS (distant metastasis, patient age, completeness of resection, local invasion and tumour size) predictive prognostic score (Figure S1), which supports an upregulation of telomerase components in tumours with a high risk of worse outcome.

Kaplan–Meier (log‐rank) test and univariate Cox regression analysis of identified genes and the known clinical prognostic markers age at diagnosis and sex^38^, ^39^ revealed that age, *TERT* and *TERC* expression were strongly associated with disease progression (*p* < .01) (Figure 1D and Figure S2A). Multivariate Cox regression model analysis determined that these three covariates harboured independent impacts on the prognosis of disease progression (age at diagnosis: HR = 1.06, 95%CI = 1.02–1.10, *p* = .002; *TERT* expression: HR = 3.19, 95%CI = 1.14–8.96, *p* = .027; and *TERC* expression: HR = 1.83, 95%CI = 1.27–2.63, *p* = .001) (Figure 1D). Finally, ROC curve analysis for the prediction of disease progression showed that the combination of the three covariates holds a discriminatory power (AUC) equal to .877 (95%CI: .79–.96, *p* = 6.6 × 10^−7^) (Figure 1E), highlighting its potential utility as a prognostic test from presurgical biopsies. Notably, multivariate Cox regression analysis for the widely used prognostic predictor AJCC stage, *TERT* expression and *TERC* expression generated a prognostic model, including *TERC* expression and AJCC stage (Figure S2B). ROC analysis for disease progression also showed that *TERT* and *TERC* expression analysis added a predictive value to the AJCC stage variable, being the combination of AJCC stage and *TERC* expression the most significant (Figure S2C). Therefore, these results reinforce the clinical applicability of the expression analysis of these two genes in surgical samples.

### 3.2 *TERT* gene *locus*‐related prognostic hallmarks co‐occurred in more advanced thyroid tumours and are associated with gene expression

**FIGURE 2:**
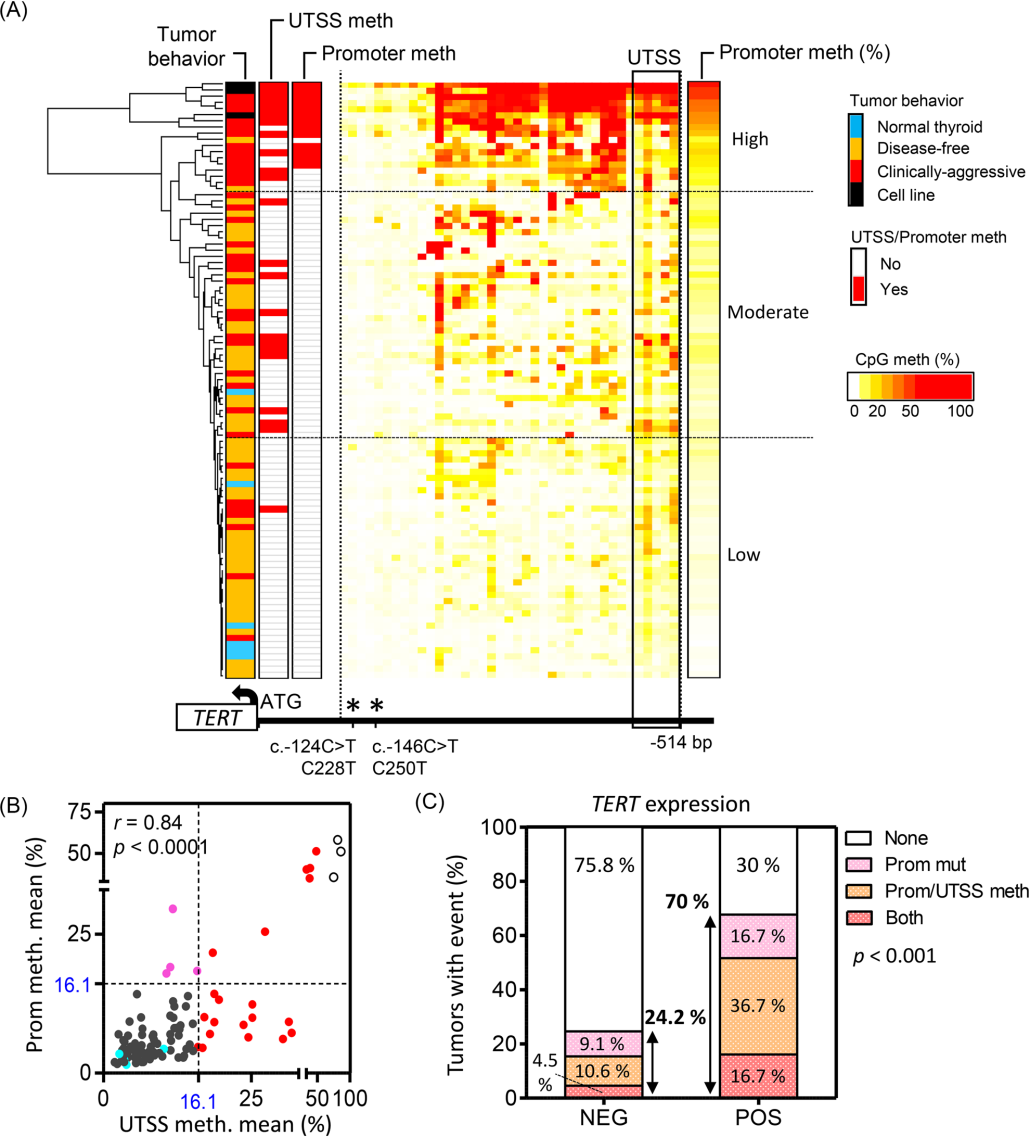
*TERT* promoter mutations and methylation are significantly associated with *TERT* expression in thyroid tumours. (A) Heat map of unsupervised clustering for thyroid specimens according to the methylation percentage of 39 CpG sites within *TERT* hypermethylated oncological region (THOR, *n* = 29) and the proximal region to the transcription start site (*n* = 10) in *TERT* promoter. Methylation mean percentage of 16.1% for either UTSS (upstream of the transcription start site) region, comprising 5 CpGs (within the black box), or for total analysed promoter region (39 CpG sites) was used to dichotomize samples in methylated (red) or non‐methylated (white). Mean promoter methylation % (promoter meth %, 39 CpG sites) is also represented using the CpG methylation (%) colour scale. Horizontal dashed black lines divide the three different *TERT* promoter methylation patterns: high, moderate and low (arbitrary thresholds). A scheme, including analysed promoter region and the start codon (ATG), is represented below the heat map, asterisks highlight *TERT* promoter mutation hotspots. (B) *XY* plot showing the correlation between the methylation of UTSS and total analysed promoter region in analysed thyroid specimen series (89 tumours, 6 normal thyroids and 3 thyroid cell lines). Nine tumours with available UTSS data were excluded from the analysis due to incomplete promoter methylation profile. Pearson correlation (*r*) analysis and two‐tailed *p*‐values are included. Blue dots: normal thyroids; empty dots: cell lines; red dots: tumour with UTSS methylation ≥16.1; pink dots: tumour with global promoter methylation ≥16.1% and UTSS <16.1%; grey dots: tumours with UTSS methylation <16.1%. (C) Stacked bar graph representing the percentage of tumours either negative (NEG) (*n* = 65) or positive (POS) (*n* = 31) for *TERT* expression with promoter mutation or/and promoter hypermethylation. A total of 96 tumours, 89 with complete profile for analysed *TERT* promoter region (*n* = 39 CpGs) and 7 with partial profile are included in the analysis. *p*‐Value of the two‐sided Fisher's exact test to determine the association between the presence of promoter methylation and mutations and *TERT* expression is shown.

To refine the prognostic value of *TERT*‐expression‐related alterations in thyroid cancer, we analysed the methylation and mutation *status* of *TERT* promoter in the whole series (*n* = 106) by next‐generation‐sequencing and determined *TERT‐locus* copy number for a subset of 31 tumours by SNP‐array. First, *TERT* promoter methylation analysis comprised 39 CpG sites within the −514‐ to −120‐bp region from the start codon (ATG), which included the 5 CpGs (−514 to −482 bp) of the UTSS region defined by Lee et al.^17^ (Figure 2A). As previously described, the correlation analysis of the methylation levels between total analysed region of *TERT* promoter and UTSS region was acutely significant (Pearson *r* = .839, *p* < .0001) (Figure 2B), and tumours either with UTSS region or with a total promoter region methylation average superior to established threshold of 16.1% were considered *TERT* promoter methylated. Notably, 50% of clinically aggressive tumours (21/42) of our series were positive for *TERT* promoter methylation as they exceeded the 16.1% cut‐off. In addition, unsupervised analysis of *TERT* promoter methylation profiles pointed to 3 major patterns according to tumour clustering, clusters proximity and profiles similarity: (1) high methylation pattern, significantly enriched in clinically aggressive tumours (13/15, 86.7%, Fisher exact test *p* = .0001) and the three thyroid cell lines used as positive control of *TERT* promoter methylation (CAL‐62, 8505C and Nthy‐ori 3‐1)^17^; (2) moderate methylation pattern, which included clinically aggressive tumours (16/39, 41%) and long‐term disease‐free associated tumours (23/39, 59%) in closer proportions; and (3) low methylation pattern, significantly enriched in disease‐free associated tumours (27/34, 79.4%, Fisher exact test *p* = .0035) and normal thyroids (5/6, 83.3%) (Figure 2A), suggesting a gradually increasing methylation of *TERT* promoter during tumour progression.

Regarding *TERT* promoter mutations, this event was significantly more frequent in clinically aggressive tumours (45.7%, 21/46) than in disease‐free tumours (3.4%, 2/58) (*p* < .0001, Fisher exact test), being the frequencies of c.‐124C>T (C228T) and c.‐146C>T (C250T)‐specific mutations 87% (20/23) and 13% (3/23), respectively. The presence of *TERT* promoter mutations showed a superior specificity for tumour clinical aggressiveness than the methylation of *TERT* promoter, as only 3.4% (2/58) against 10.9% (6/55) of disease‐free tumours were positive for *TERT* promoter mutations and promoter methylation, respectively (Figure S3A,B). Copy number gains at *TERT‐locus* were also more frequent among clinically aggressive tumours (29.4%, 5/17) than in disease‐free tumours (7.1%, 1/14), but the differences did not reach statistical significance, probably due to sample size (Figure S3B).

Notably, co‐occurrence of *TERT* promoter mutation and methylation was exclusively observed in clinically aggressive tumours and were more frequent in advanced histology tumours (Figure S3A,C, and Table S1, ^26^). Copy number analysis reinforced this observation, as five of the six thyroid tumours with copy number gains at *TERT locus* were anaplastic thyroid tumours or differentiated with an undifferentiated component. In addition, four out of these five showed co‐occurrence of *TERT locus* gains and *TERT* promoter alterations (Figure S3A and Table S1, ^26^). Overall, these results indicate that the accumulation of pathogenic alterations at *TERT‐locus* is associated with more advanced thyroid cancer disease.

The 70% (21/30) of tumours with *TERT* expression held at least one pathogenic alteration at *TERT* promoter, being promoter methylation more frequent than the presence of promoter mutations (53.3%, 16/30 and 33.3%, 10/30, respectively) (Figure 2C and Figure S3A). Among the nine *TERT* expressing tumours negative for promoter mutations and methylation, six corresponded to patients with excellent response to the primary treatment with a median follow‐up of 120 months (72–144‐month range). Histological examination and subsequent immunohistochemical analysis of LCA CD45 revealed that four out of the five available tumour specimens showed lymphocyte infiltration (Figure S4), which has been recently associated with tumour cell‐unspecific positivity for *TERT* expression.^20^

### 3.3 Telomere length is associated with co‐occurrence of immortalization hallmarks in thyroid tumours

**FIGURE 3:**
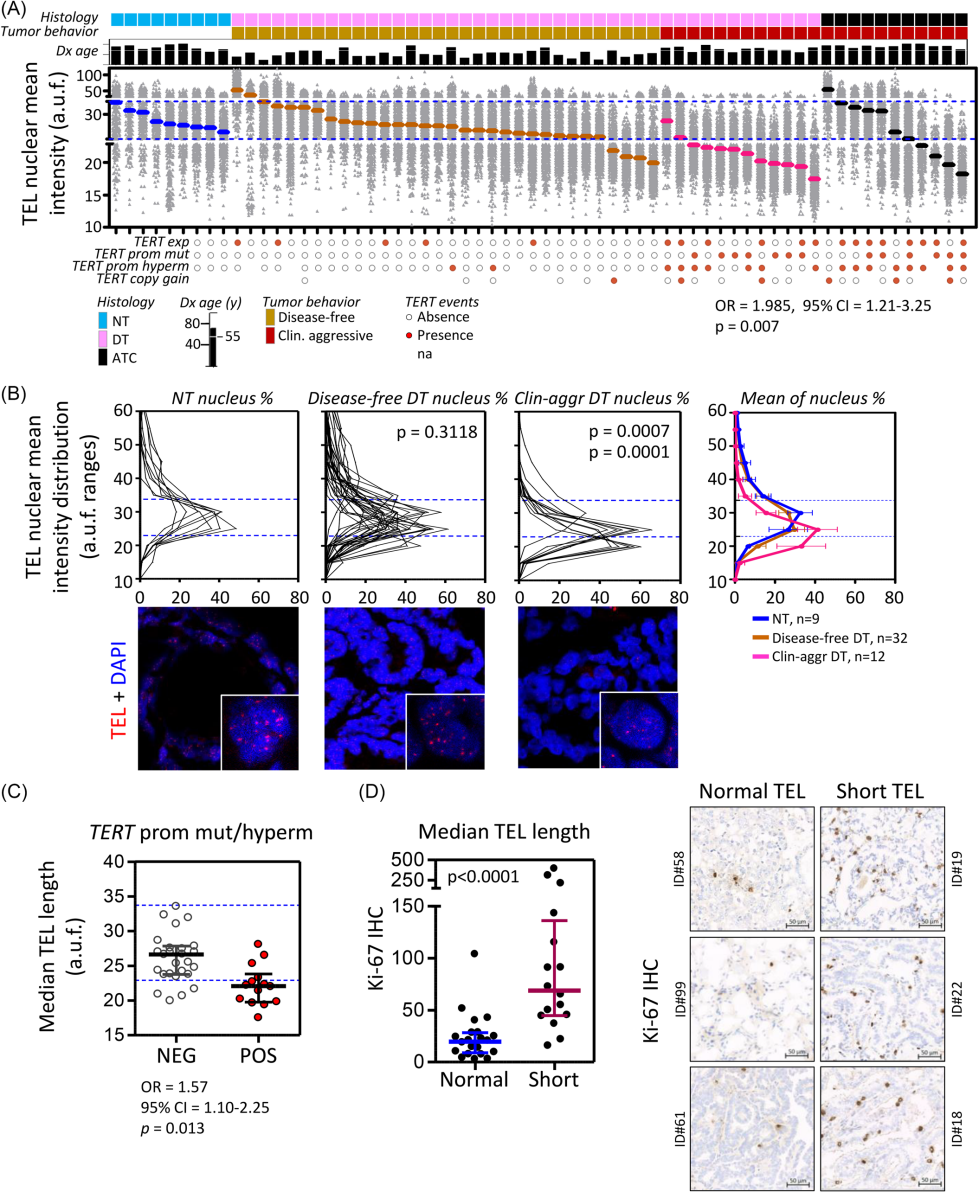
Tumour telomere length is associated with the clinical behaviour of the disease, tumour proliferation and the presence of pathogenic alterations at *TERT‐locus*. (A) Scatter dot plot representing the telomere (TEL) nuclear mean intensities for a series of thyroid tumour (*n* = 55) and normal tissues (*n* = 9). a.u.f., arbitrary units of fluorescence. Each grey dot represents the telomere mean intensity of a nucleus. Median telomere lengths of each specimen are represented by a thick horizontal line coloured according to histology class. Bright blue lines represent the upper (34 a.u.f.) and lower thresholds (23 a.u.f.) of normal telomere length range established as the mean telomere lengths ±2*SD of analysed normal thyroids (NT). Binary logistic regression analysis results for clinical behaviour and tumour median telomere length adjusted for the covariate age at diagnosis (Dx age, years (y)) are shown. OR, odd ratio of persistent/recurrent disease in patients with short telomere length tumours; 95%CI, 95% confidence interval; *p* = *p*‐value. DT, differentiated tumour; ATC, anaplastic thyroid cancer. (B) Distribution plots of nuclear intensity of TEL. Two‐tailed Mann–Whitney test *p*‐values comparing the mean nuclear intensities between NT and disease‐free thyroid tumours (TC), NT and clinically aggressive DT, disease‐free DT and clinically aggressive DT are shown below the corresponding graph. A representative TEL FISH (fluorescence in situ hybridization) image of the three different tissue classes are included below the respective plots. (C) Scatter dot plot of tumour telomere lengths (median of TEL intensity) according to tumour status (NEG/POS) for *TERT* promoter mutations or/and hypermethylation. Each dot represents a tumour. Bright blue dashed lines represent the upper (34 a.u.f.) and lower thresholds (23 a.u.f.) of normal telomere length range established as the mean telomere lengths ±2*SD of a set of nine NT. Binary logistic regression analysis results for *TERT* promoter mutations and tumour median telomere length adjusted for the covariate age at diagnosis are shown. OR, odd ratio of occurrence of *TERT* promoter mutations in tumours with short telomere lengths; 95%CI, 95% confidence interval; *p* = *p*‐value. (D) Plot representing the automatic image analysis results using ZEISS ZEN 3.1 software of Ki‐67 immunostaining for complete sections of differentiated thyroid tumours with either normal telomeric length (*n* = 22, normal) or short telomere length (*n* = 16, short). Dots represent Ki‐67 positive area relative to total area (×10 000). Median and interquartile ranges for ‘normal’ and ‘short’ tumours are represented. Mann–Whitney test *p*‐value is shown. On the right, representative images of Ki‐67 immunostaining for thyroid tumours with either normal or short telomere lengths are included.

To characterize telomere length of thyroid tumours and evaluate its association with tumour clinical behaviour and aforementioned prognostic hallmarks, we performed telomere Q‐FISH analysis in a set of 55 tumours using a telomere‐specific probe.^40^ We established the short telomere length cut‐off in 23 a.u.f. (arbitrary units of fluorescence), which corresponded to the mean value −2*SD of the telomere intensities of a set of normal thyroids (*n* = 9) from individuals with ages between 50 and 82 years (median = 67‐year old). Although 91.7% (11/12) of clinically aggressive differentiated carcinomas exhibited short telomere lengths (mean telomere intensity ≤ 23 a.u.f.), only 15.6% (5/32) of disease‐free associated tumours reached the established short telomere cut‐off (*p* < .0001, Fisher exact test) (Figure 3A and Table S1, ^26^). Tumour telomere median length significantly correlated with the percentage of shortest telomeres (Pearson *r* = −.632, *p* < .0001) (Figure S5A). Accordingly, telomere length mean distribution of clinically aggressive tumours was significantly shifted towards shorter telomere lengths compared to normal thyroids (*p* = .0007) and disease‐free tumours (*p* = .0001) (Figure 3B).

The most advanced histologies (ATC) showed a heterogeneous median length with four tumours fitting short telomeres cut‐off (4/11, 36.4%), and the other three with large and ultra‐bright telomere FISH signals characteristic of alternative lengthening of telomeres (ALT)‐associated telomeric *foci*.^41^ ALT‐phenotype was exclusively observed in these specimens (Figure S5B), which may be related to the acute transformation degree of these carcinomas, acquired mesenchymal phenotype and aneuploidy. In differentiated tumours, telomere shortening was significantly associated with the presence of *TERT* promoter mutations and/or methylation (Figure 3C) with the statistical analysis being adjusted for age at diagnosis (OR = 1.57; 95%CI = 1.10–2.25; *p* = .013), and it correlated with the expression of *TERC* (Pearson *r* = .335, *p* = .0131) and *DKC1*, although not significant for the latest (Pearson *r* = .200, *p* = .0965) (Figure S5C). Moreover, differentiated tumours with short telomeres were significantly more positive for the nuclear immunostaining of the proliferation marker Ki‐67 than those tumours with normal telomere lengths (2‐tailed Mann–Whitney test *p* < .0001) (Figure 3D), supporting the replicative‐dependent shortening of the telomeres in thyroid tumours. Methylation of *TERT* promoter (methylation average of the 39 CpG sites within the −514‐ to −120‐bp region from ATG site >16.1%) and co‐existence of pathogenic events at *TERT locus* and gene expression were attributes mainly limited to short telomere tumours in differentiated carcinomas (Figure S5D and Figure 3A), suggesting that telomere shortening facilitates the occurrence of structural and transcriptional alterations related to *TERT locus*.

### 3.4 Telomere shortening is associated with over‐expression of subtelomeric genes and chromatin reorganization

**FIGURE 4:**
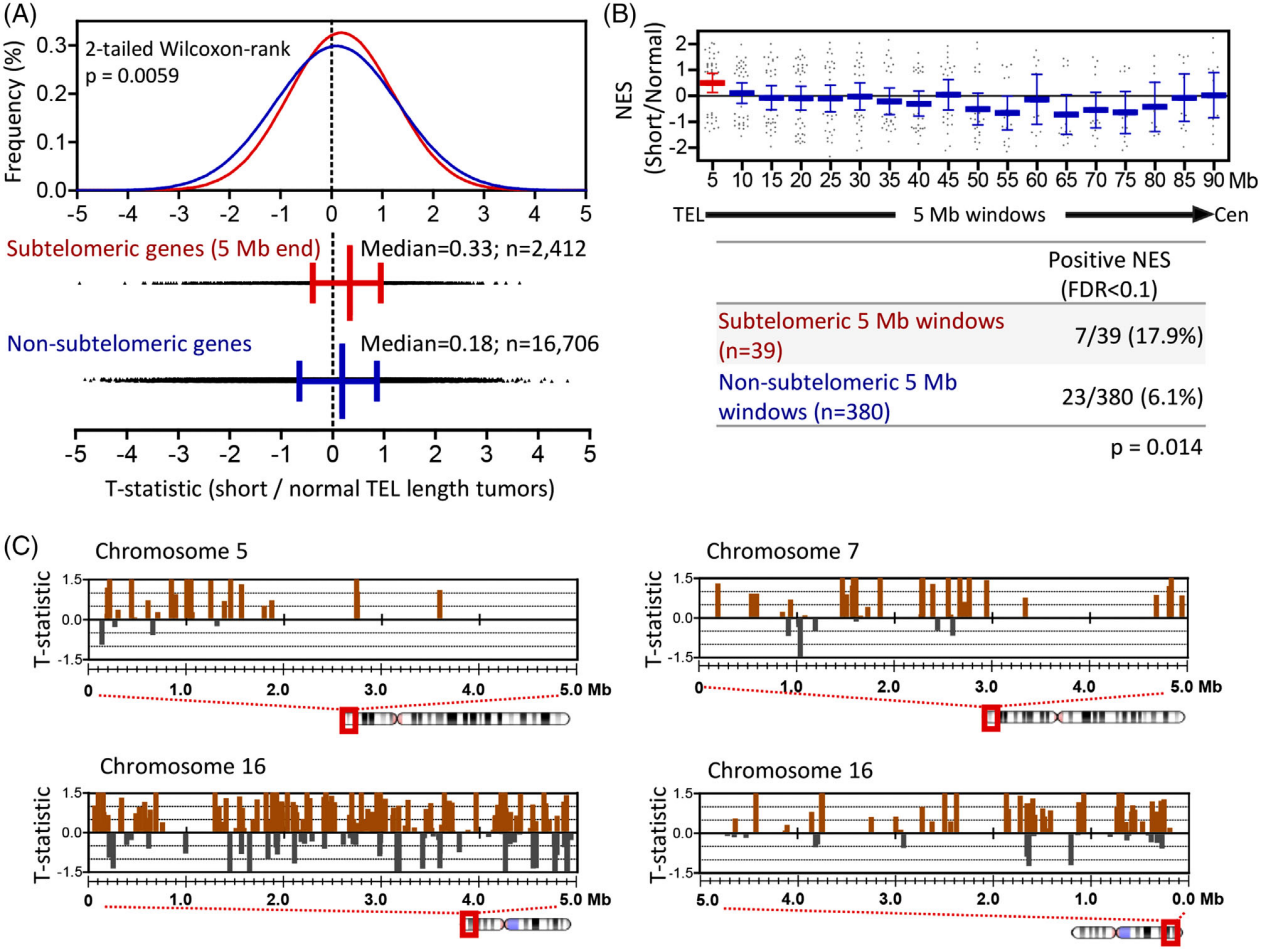
Telomeric shortening is associated with a higher expression of subtelomeric genes. (A) Frequency distribution of moderated *T*‐statistic from gene expression comparison between short and normal TEL length tumours for genes mapped within (subtelomeric) or out (non‐subtelomeric) of the 5‐Mb‐ends of the chromosomes (red and blue, respectively). Two‐tailed Wilcoxon matched‐pairs signed rank‐test *p* value is shown. Median of both distributions are included below. *n*, number of genes in each set. (B) Scatter dot plot of normalized enrichment scores (NES) for genes within 5‐Mb genomic windows from telomeres (TEL) to centromeres (Cen). Only 5‐Mb windows with at least 15 gene expression data were considered in the analysis. NES are calculated from the moderated *T*‐statistic of gene expression comparison between short telomere and normal telomere length tumours. Positive NES values indicate an overrepresentation of the respective genomic window genes among upregulated genes in short telomere tumours compared to those with normal telomere length. Red bars represent mean NES value with 95% confidence interval (CI) of the subtelomeric regions; blue bars indicate mean NES values with 95%CI of non‐subtelomeric regions according to their distance from the telomeres. Contingency table and Fisher's exact test *p*‐value comparing the number of 5‐Mb windows with significant enrichment scores (FDR < .1) between subtelomeric regions (*n* = 39) and non‐subtelomeric regions (*n* = 380) are included. (C) The 5‐Mb subtelomeric regions of indicated chromosome arms. Brown bars, genes upregulated in short telomere tumours; grey bars, downregulated genes. The bar position reflects the gene location on the chromosome. The height of the bars is proportional to the moderated *T*‐statistic of expression between short and long telomeric length tumours.

To determine whether telomere shortening in thyroid tumours delineates gene expression of subtelomeric regions as described in yeast and mammalian cells,^22^, ^25^ we performed RNA sequencing in a subset of tumours (*n* = 15) and compared gene expression profiles of subtelomeric and non‐subtelomeric genes between short telomere tumours (*n* = 10) and normal telomere tumours (*n* = 5). We defined as subtelomeric genes, those located within the 5‐Mb windows adjacent to the telomeres, which allowed us to include a robust number of genes for subtelomeric and non‐subtelomeric regions (Figure 4A). In both datasets, the median gene expression distribution was shifted from zero, indicating genome‐wide increased expression in tumours with short telomeres compared to tumours with normal length telomeres. However, the increase in gene expression was superior in subtelomeric than in non‐subtelomeric regions (median = .33 and .18, respectively), being this difference statistically significant (2‐tailed Wilcoxon matched‐pairs signed rank‐test *p* = .0059) (Figure 4A). Similarly, gene set enrichment analysis (GSEA) of the genome segmented into 5‐Mb windows from telomere adjacent region to centromere showed that although 17.9% (7/39) of the subtelomeric windows were significantly enriched (FDR < .1) of upregulated genes in short telomere tumours, only 6.1% (23/380) of non‐subtelomeric 5‐Mb windows showed this trait. Significant non‐subtelomeric windows did not accumulate at specific positions in the genome (Figure 4B). Functional annotation analysis indicated that there was an enrichment of telomerase‐related terms among genes at 5‐Mb chromosome ends (Figure S6A). Specific GSEA for subtelomeric genes determined that the 5‐Mb ends of chromosomes 7p, 5p, 16p and 16q were the most significantly enriched of upregulated genes in short telomere tumours compared to normal telomere length tumours (FDR < .05), being *TERT* located 1.2 Mb from chromosome 5p end (Figure S6B,C). *T*‐Statistic values from gene expression comparison of short versus normal telomere length tumours for each of the subtelomeric genes at these chromosomal *loci* were extensively positive, sustaining that these sites are transcriptionally more active in short telomere tumours (Figure 4C). Finally, as we observed a significant association between telomere shortening and high expression of the proliferation marker Ki‐67 (Figure 3D), and high correlation between Ki‐67 protein and mRNA expression abundancy (Figure S7A), we used this later marker (*MKI67* gene expression) to select TCGA tumours with putative short telomeres (Figure S7B). We found an upregulation of 5p‐end genes including *TERT* in TCGA tumours with high *MKI67* expression, supporting our findings (Figure S7C).

**FIGURE 5:**
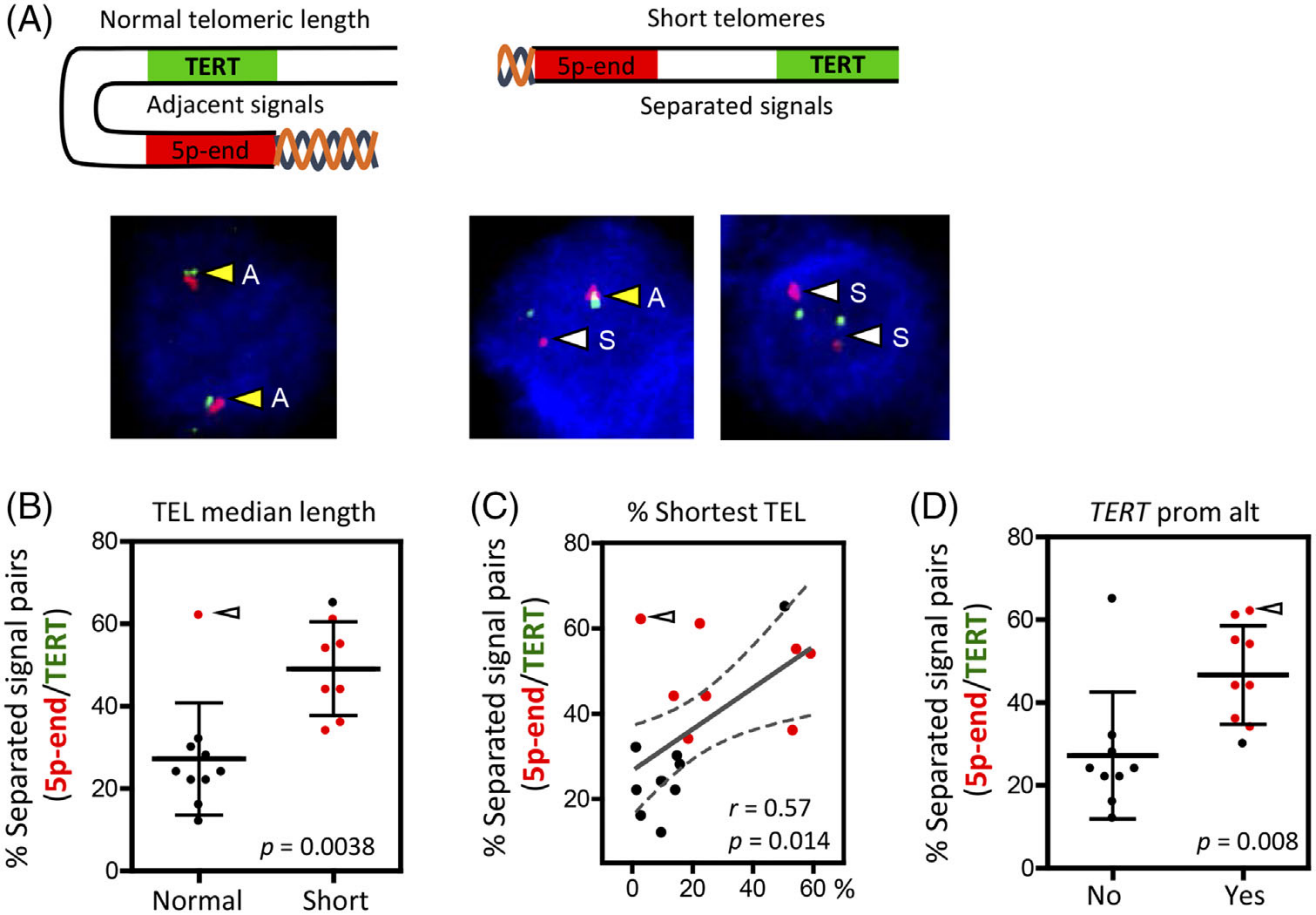
Telomere shortening is associated with configuration changes in the chromatin of the chromosome 5p.ter. (A) Upper panel, scheme of 5p‐chromosome‐end representing the design of the fluorescence in situ hybridization (FISH) assay. The red probe stains the sub‐telomeric region of chromosome 5p and the green probe stains *TERT‐locus*. Probe signals were analysed with a fluorescence microscope for at least 200 cells/tumour and classified as adjacent or separated events according to signal proximity. Down panel, representative co‐FISH images of thyroid tumour nuclei. Yellow arrows highlight adjacent signals (A) and white arrows highlight separated signals (S). (B) Scatter plot of the percentages of separated signals for normal length tumours (*n* = 10) and short telomere tumours (*n* = 8). Mean values with SD are represented for each tumour group. *p*‐Value of two‐tailed Mann–Whitney *t*‐test is shown. Each dot represents a thyroid tumour (in red: clinically aggressive tumours; in black: disease‐free tumours). (C) Linear regression plot for the percentage of shortest telomere (telomere FISH signals < 20 a.u.f.) (*x*‐axis) and 5p‐end/*TERT* separated events (*y*‐axis). Red dots represent clinically aggressive tumours; black dots represent disease‐free tumours. Pearson *r* coefficient and *p*‐value are shown. Dashed line curves indicate 95% confidence intervals. Red dots: clinically aggressive tumours; black dots: disease‐free tumours. (D) Scatter plot of 5p‐end/*TERT* separated signals percentage in tumour without (No, *n* = 9) or with (Yes, *n* = 9) *TERT* promoter pathogenic mutations or/and hypermethylation (*TERT* prom alt). *p*‐Value of two‐tailed Mann–Whitney *t*‐test is included. Mean values are plotted with SD for each tumour group. Red dots in B, C and D: clinically aggressive tumours; black dots in B, C and D: disease‐free tumours. White arrow in B, C and D point to poor prognosis tumour ID#6

To validate the cause‐effect relationship between short telomere length and gene expression up‐regulation at 5p subtelomeric region, we examined chromosome organization by a co‐FISH assay for 5p‐end (.075 Mb from the telomere, red probe) and *TERT locus* (1.2 Mb from the telomere, green probe) (Figure 5A). Proximity analysis of the signals across a set of short telomere (*n* = 8) and normal telomere length tumours (*n* = 10) showed that while the median percentage of separated probes was 24% (IQR: 20.5%–30.5%) in normal length tumours, the percentage of separated signals significantly increased to a median value of 49% (IQR: 38%–59.5%) in short telomere tumour (*p* = .038) (Figure 5B). Distance analysis between probes using high‐resolution confocal microscope (Leica SP8) furtherly confirmed that median distance between 5p‐end and *TERT* signals was superior in short telomere tumours (*n* = 3) than in normal telomere length tumours (*n* = 3) (Figure S8). Linear regression analysis confirmed the significant correlation between the percentage of separated signal pairs and percentage of shortest telomere lengths in thyroid tumours (Pearson *r* = .57, *p* = .014) (Figure 5C), reinforcing the hypothesis that progressive telomere shortening induces chromosome reorganization at 5p‐subtelomeric region. In‐line with this, the presences of *TERT* promoter mutations and/or methylation were significantly associated with increased percentages of separated signal pairs (*p* = .008) (Figure 5D). Notably, the only poor prognosis tumour with a median telomere length within the range of normal thyroids (ID#6), and with *TERT* promoter methylation and *TERT* expression, showed a high percentage of separated signal pairs (Figure 5B–D), suggesting that telomere length of chromosome 5p was shortened. The results point to the co‐FISH assay for the 5p‐end and TERT locus as a promising cytogenetic tool for determining thyroid cancer prognosis.

## 4 DISCUSSION

Studies that simultaneously address the complexity of thyroid cancer disease and immortalization mechanisms are needed to establish a sequence of events in tumour progression, identify the molecular alterations involved in the early stages of the immortalization process and refine the prognostic value of known markers. Our study compiles a series of 106 tumours that embraces the clinical heterogeneity of the disease and exhaustively characterizes molecular alterations related to immortalization process as well as determines tumour telomere lengths. We identified five genes of the telomerase holoenzyme complex protein upregulated in aggressive tumours. Among identified genes, *TERT* expression and *TERC* overexpression showed independent predictive impacts on progression free survival, which, together with the age at diagnosis, held a moderate discriminatory power for distinguishing tumours from patients with excellent response after initial treatment (median follow‐up >8 years) from those with increased morbidity and associated high health care cost (evidence of disease, recurrence or/and disease specific mortality during the follow‐up). This finding is in consonance with late 1990s discoveries, in which it was shown that the expression of the RNA component of telomerase *TERC*, constitutively expressed in somatic cells, was upregulated early in tumour progression, whereas *TERT* expression was only activated in late stages,^42^ indicating that both genes might have a predictive value in the clinical setting. A more recent study using transcriptomic data of 6835 tumours across 31 cancer types determined a striking association between *TERC* upregulation and *TERT* expression,^8^ supporting previous findings in experimental models and our results in thyroid tumour specimens, in which the analysis of both genes improves prognostic predictive performance of each gene expression separately. With regard to the mechanisms leading to the upregulation of these two genes, respective gene *locus* amplifications have been previously associated with their aberrant expression in cancer.^8^ However, copy number analysis performed herein sustained that genomic gains or losses at these and other telomerase complex genes’ *loci* mildly explains such expression patterns observed in our tumour series (Figure S9). Thus, other aberrant mechanisms should be driving deregulation of our candidate genes in thyroid cancer, as it has been widely described for *TERT*.^12^, ^13^, ^14^, ^15^, ^16^, ^17^, ^18^

*TERT* expression in immortalized cells can be intermittent or slightly activated,^43^ challenging its detection in the clinical setting. Thus, tumour *status* for *TERT* promoter mutations, a highly cancer‐specific *TERT* expression‐related alteration, has been widely used to predict disease outcome in certain cancers.^13^, ^44^ Yet, each cancer can exhibit a variety of *TERT* expression‐related events with different prevalence pattern across cancer types,^7^, ^8^ which hampers the translation of the molecular knowledge into a prognostic tool for daily clinical practice. Specifically, the methylation of *TERT* promoter has become a hallmark of *TERT* re‐expression in multiple tumour types. Although the mechanistic details are not yet fully understood, methylation of *TERT* promoter seems to impair the binding of transcriptional repressors.^45^ In thyroid cancer, our study indicates that the methylation of *TERT* promoter is as prevalent as *TERT* promoter mutations among clinically aggressive tumours (50% vs. 45.7% respectively), although with less specificity for disease prognosis. The high percentage of clinically aggressive tumours that we observed showed promoter methylation, but the absence of promoter mutations (60%, 12/20) denotes the limited prognostic predictive value of testing only *TERT* promoter mutations, which is in consonance with previous studies.^17^, ^46^ On the other hand, tumour‐lymphocytic infiltrates could lead to a misinterpretation of *TERT* expression results in some particular cases, as this cell lineage can maintain *TERT* expression after differentiation.^20^ In fact, this feature could explain the positivity of *TERT* expression of most of the tumours in our series with an exceptional response to primary treatment, and without other molecular or clinical evidence related to poor prognosis. Thus, our results underscore the need to consider *TERT* promoter methylation in screening tools for the stratification of thyroid tumours according to their malignant potential and to report *TERT* gene expression results along with tumour cellular composition. Overall and despite the previously mentioned limitations, our study supports that a comprehensive analysis of the pathogenic mechanisms that reactivate *TERT* expression in differentiated cells together with *TERC* expression may constitute a prediction model for detecting patients at high risk to develop progressive disease. Particularly, our study highlights the clinical applicability of the analysis of TERC and *TERT* expression in surgical specimens, which could be extrapolated to pre‐surgical biopsies. Future studies that will delve into the clinical application of these markers are warranted.

The observed high prevalence and prognostic specificity of different expression‐related alterations at *TERT*‐*locus*, and the exclusive co‐existence of these events in our series of clinically aggressive tumours, evidences that *TERT*‐*locus* is a critical susceptibility site for tumour progression in thyroid cancer. These findings support the hypothesis that there is a stage in tumour progression from which an accumulation of these events is prone to occur. However, the mechanism that favours this scenario is uncertain. Robin et al.^24^, ^47^ demonstrated that telomeres interact with sub‐telomeric regions at distances as far as 10 Mb from the end of the chromosome, which given the location of *TERT* (1.2 Mb from the end of 5p‐chromosome) could compromise the functionality of the *locus*. As telomeres shorten with cell divisions, the looping interactions are lost and the chromatin is reorganized into an open conformation.^25^, ^48^ Therefore, we hypothesized that in thyroid tumours, telomere shortening upon proliferative pressure leads to a configuration change at *TERT* that facilitates the occurrence of pathogenic events. Our analysis of telomere lengths and proliferation marker Ki‐67 supported the replicative‐dependent shortening of telomeres in thyroid tumours. Notably, most of the tumours with co‐occurring events at *TERT*‐*locus*, and those with an acute and extensive *TERT* promoter methylation, showed short telomere lengths, fitting with our hypothesis. We also observed that one of the four tumours from disease‐free patients with long follow‐ups and short telomeres showed elevated *TERC* expression and *TERT* copy gain. Although clinically these tumours have responded excellently to primary treatment, molecularly they may have reached the immortalization stage at surgery. Therefore, tumours with striking telomere shortening but negative for *TERT* expression and analysed alterations may be at an early stage of the immortalization process,^24^, ^47^ and these patients could benefit from specific and extended follow‐up strategies.

Our transcriptomic analysis of a set of tumours further supported our hypothesis as we determined that thyroid tumours with short telomeres were more transcriptionally active at the 5‐Mb‐ends of chromosomes than normal telomere lengths tumours. This association was not equally strong among all chromosome ends, being acutely significant for specific chromosome‐ends, such as the 5p. The differences in gene regulation at chromosome ends could be dependent on the presence and number of interstitial telomere sequences around the genes located at those regions. This trait determines the formation of looping‐interactions with telomeres through shelterin‐complex and, consequently, gene regulation dependency on telomere‐length.^48^, ^49^ In‐line with these studies, our co‐FISH results of *TERT‐locus* and the subtelomeric‐end of chromosome 5p showed that telomere length determines conformation differences at this subtelomeric region, supporting that telomere shortening leads to a more relax or transcriptionally active conformation of chromosome 5p‐end in thyroid cancer. Besides *TERT*, other genes encoding telomerase holoenzyme complex proteins such as *DKC1* (Xq28), which we identified significantly upregulated in clinically aggressive tumours, and *NHP2*(5q35), are within the 5‐Mb‐ends of their respective chromosomes. Additional studies will establish whether telomere shortening is an early regulatory mechanism of their expression.

## 5 FINAL CONCLUSION

The results of this study reinforce the role of *TERT* upregulation together with other components of telomerase holoenzyme complex, such as *TERC*, as prognostic factors and preferential immortalization mechanism in thyroid cancer. Combined expression of *TERT* and *TERC* is a promising maker with a feasible applicability in the clinical setting. Although events associated with *TERT* upregulation are uncommon and unique in disease‐free associated tumours, these events are particularly frequent and co‐occur in clinically aggressive tumours, which suggests a positive selection of these attributes during cancer progression. Thyroid tumours with shorter telomeres are more proliferative than those with ‘normal‐range’ telomere lengths and exhibit a more transcriptional permissive environment in the subtelomeric regions. This feature is associated with chromatin reorganization at chromosome 5p‐end and expression of *TERT*, which is located in this region. Our results point to a step‐wise model for the immortalization process in thyroid cancer and reveal FISH‐based analysis of 5p‐end genomic region as a potential molecular cytogenetic tool to predict the prognosis of thyroid tumours.

## CONFLICT OF INTEREST

The authors have declared that no conflict of interest exists.

## Supporting information

Supporting Information

Supporting Information

Supporting Information
