# Supplementary material for: Comprehensive molecular analysis of immortalization hallmarks in thyroid cancer reveals new prognostic markers
Source: Clin Transl Med. 2022 Aug 18;12(8):e1001. doi: 10.1002/ctm2.1001 (PMC9386325; doi:10.1002/ctm2.1001)
Supplement: Supplementary file 1 — Supporting Information [file CTM2-12-e1001-s001.pdf]

Comprehensive molecular analysis of immortalization hallmarks in thyroid cancer reveals new prognostic markers.

Cristina Montero-Conde[1,2*], Luis Javier Leandro-García[1], Ángel M. Martínez-Montes[1], Paula Martínez[3], Francisco J. Moya[4], Rocío Letón[1], Eduardo Gil[1], Natalia Martínez-Puente[1,2], Sonsoles Guadalix[5], Maria Currás-Freixes[6,7], Laura García-Tobar[8], Carles Zafon[9], Mireia Jordà[10], Garcilaso Riesco-Eizaguirre[11,12], Patricia González-García[13], María Monteagudo[1], Rafael Torres-Pérez[1,14], Veronika Mancikova[1], Sergio Ruiz-Llorente[15], Manuel Pérez-Martínez[16], Guillermo Pita[17], Juan Carlos Galofré[18,19], Anna Gonzalez-Neira[2,17], Alberto Cascón[1,2], Cristina Rodríguez-Antona[1,2], Diego Megías[16], María A. Blasco[3], Eduardo Caleiras[13], Sandra Rodríguez-Perales[4], Mercedes Robledo[1,2*].

Authorship note: CMC and MR are co-corresponding authors. CMC and LJL-G are co-first authors.

SUPPORTING INFORMATION

Table S2

Figures S1-9

Methods Table S2

Table S2. Clinicopathological characteristics of patients with differentiated thyroid cancer (DTC) dichotomized into disease-free and clinically aggressive disease.

	Disease-free (n=55)[a]	Clinically aggressive (n=26)[b]
Variables (categories)	n (%)	n (%)
Age		
<55	41 (74.5)	12 (46.2)
≥55	14 (25.5)	14 (53.8)
Sex		
Female	41 (75.9)	17 (65.4)
Male	13 (24.1)	9 (34.6)
Primary tumor (T)		
T1	17 (30.9)	4 (15.4)
T2	20 (36.4)	5 (19.2)
T3	18 (32.7)	13 (50.0)
T4	0 (0.0)	2 (7.7)
TX	0 (0.0)	2 (7.7)
Regional lymph node metastases at dx (N)		
N0	19 (34.5)	5 (19.2)
N1	15 (27.3)	19 (73.1)
NX	21 (38.2)	2 (7.7)
Distant metastases at dx (M)		
M0	55 (100.0)	5 (19.2)
M1	0 (0.0)	19 (73.1)
MX	0 (0.0)	2 (7.7)
AJCC (prognostic groups) [a]		
I	49 (89.1)	2 (7.7)
II	6 (10.9)	11 (42.3)
III	0 (0.0)	1 (3.8)
IV	0 (0.0)	10 (38.5)
not available	0 (0.0)	2 (7.7)
Extent of surgery		
Total thyroidectomy	28 (50.9)	4 (15.4)
Total thyroidectomy and lymphadenectomy	27 (49.1)	22 (84.6)
Radioiodine therapy		
Yes	53 (96.4)	23 (88.5)
No	0 (0.0)	1[c] (3.8)
Unknown	2 (3.6)	2[d] (7.7)

[a] Excellent reponders to initial treatment (structural and biochemical complete response), no evidence of disease, recurrence, or disease specific mortality along the follow-up.

[b] Clinically aggressive tumors were those from patients who showed persistent or progressive disease at the latest available follow-up date.

[c] The patient died 6 months after surgery.

[d] DTC with poorly differentiated component with no available follow-up data.

Figure S1

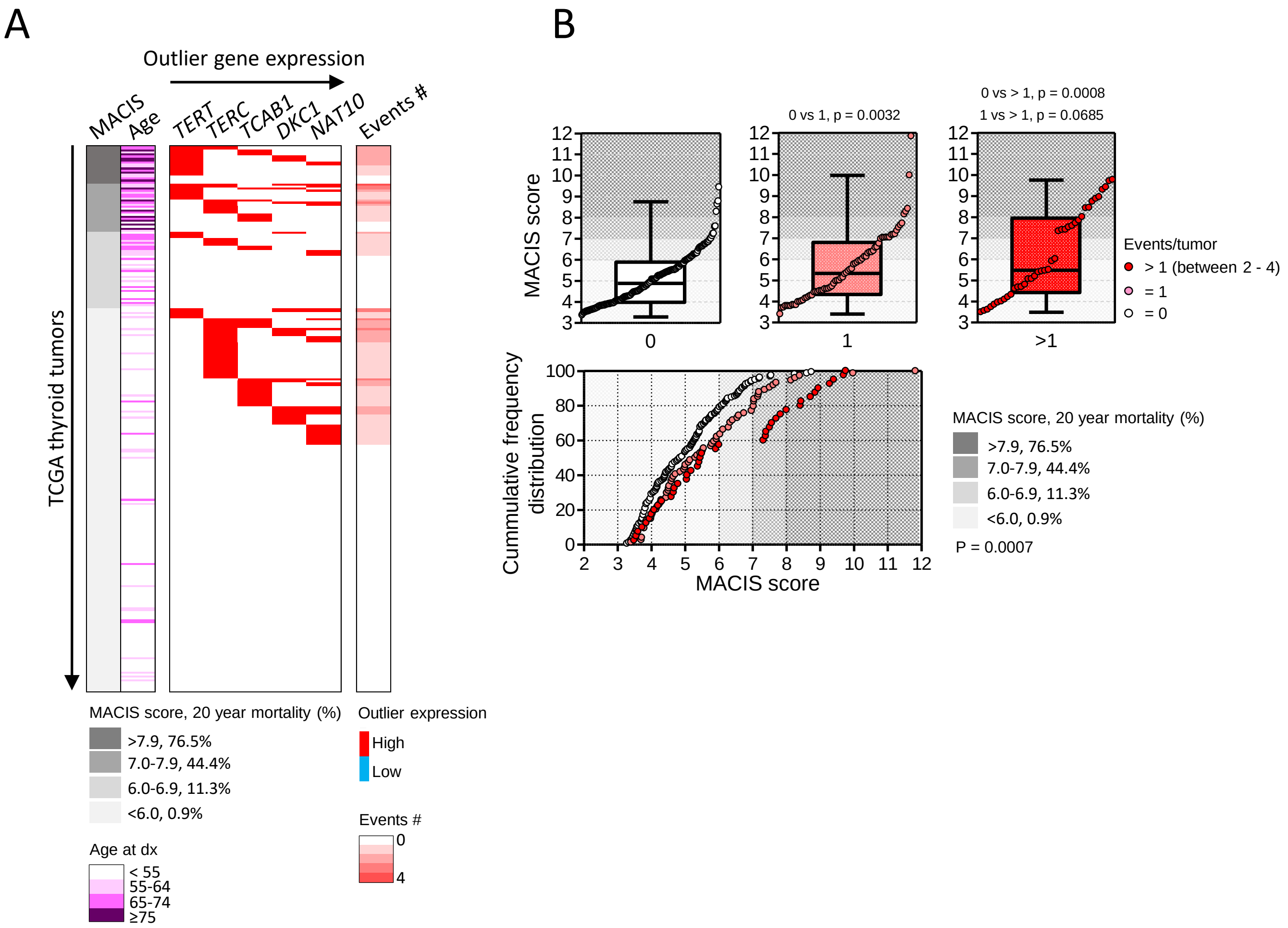

Figure S1. **TCGA thyroid tumors with a higher MACIS prognostic score accumulate gene expression outliers of telomerase holoenzyme complex genes. A.** Outlier gene expression print of TCGA thyroid tumor series for the 5 candidate genes of telomerase complex. Gene expression median and interquartile ranges (IQR) of tumors with a MACIS score < 6.0 (reference tumor set) were used to establish the outlier gene expression cutoffs. Bright red highlights tumors with outlier expression (> Median + IQR) for *TERC*, *TCAB1*, *DKC1* and *NAT10*. For *TERT* expression, tumors with normalized counts (RNA Seq V2 RSEM) > 1 were considered *TERT* expression outliers, as outlier gene expression cutoff was 0. Tumors are ranked according to MACIS score categories and their associated 20-year cause-specific mortality percentages. Events # represents the number of gene expression outliers for indicated gene set for each tumor. Only TCGA tumors with a MACIS score value and cancer cell purity >60% were considered in the analysis (n = 272). Age stands for age at diagnosis (dx). **B.** Distribution of MACIS prognostic score for the TCGA thyroid carcinoma series according to the number of expression outliers (events) of the identified gene set. Upper panel shows Tukey Whiskers plots for MACIS score values of TCGA tumors with either none (n = 129), 1 (n = 91) or more than 1 (n=52) gene expression outliers. One-tailed Mann-Whitney test p-values are shown. Lower panel shows cumulative frequency distribution of MACIS score values for TCGA thyroid tumor series stratified by the number of gene expression outliers. Background colors highlight the 20-year cause-specific mortality percentages associated with each MACIS score category. Kruskal-Wallis p-value is shown. TCGA RNAseq and clinicopathological data were downloaded from the cBioPortal (RNA Seq V2 RSEM, www.cbioportal.org) and UCSC Xena web-tool (http://xena.ucsc.edu; TCGA Thyroid Cancer (THCA) study).

Figure S2

A

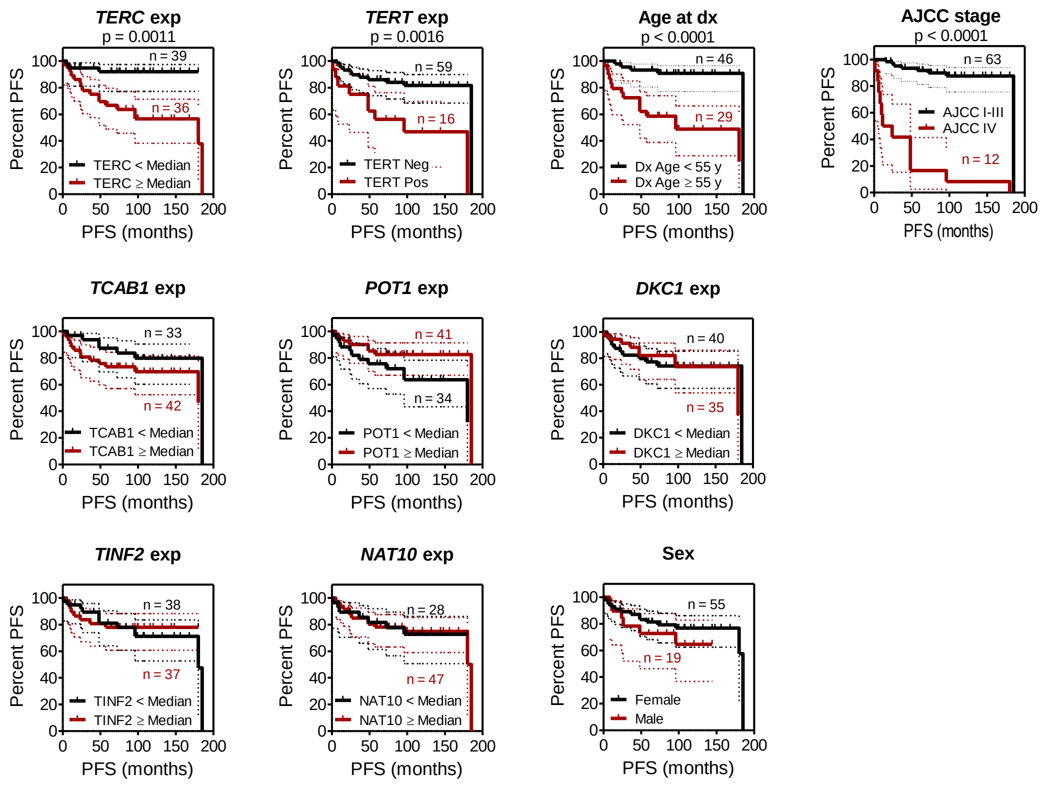

B

	Covariables	HR	95% CI	*p*-value
Univariate (PFS)	**AJCC (I-III / IV)**	18.04	6.90 - 47.16	$3.6x10^{-9}$
	TERT	3.85	1.56 - 9.50	$3.4x10^{-3}$
	TERC	1.43	1.11 - 1.86	$6.4x10^{-3}$
Multivariate (PFS)	**AJCC (I-III / IV)**	15.95	6.03 - 42.20	$2.4x10^{-8}$
	TERC	1.38	1.01 - 1.89	$4.2x10^{-2}$

C

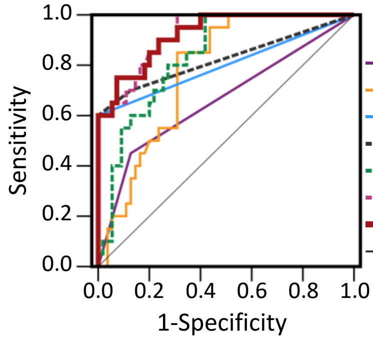

	AUC	95% CI	*p*- value
TERT	0.661	0.51 - 0.81	$3.3x10^{-2}$
TERC	0.775	0.67 - 0.88	$2.9x10^{-4}$
AJCC	0.800	0.66 - 0.94	$7.7x10^{-5}$
AJCC / *TERT*	0.825	0.69 - 0.96	$1.9x10^{-5}$
TERT / *TERC*	0.835	0.74 - 0.93	$9.8x10^{-6}$
**AJCC / *TERC***	**0.924**	**0.86 - 0.98**	$\mathbf{2.3x10^{-8}}$
**AJCC / *TERT* / *TERC***	**0.924**	**0.86 - 0.99**	$\mathbf{2.4x10^{-8}}$
Reference line			

Figure S2. **A.** Progression-free survival (PFS) Kaplan-Meier curves of thyroid cancer patients with available follow-up data (n = 75, number of patients with progressive disease = 20) for indicated variables. Patients were dichotomized into 2 groups according to the indicated thresholds. Only log-rank (Mantel-Cox) analysis significant *p*-values are included. *P*-values of variables with log-rank test p-value <0.05 are shown; Median: gene expression median for analyzed tumors (n = 75); Dx age; diagnostic age. Dashed line curves indicate 95% confidence intervals. **B.** Univariate and multivariate Cox regression analysis of progression-free survival for the variables AJCC stage, TERC and TERT expression (n = number of patients; Event (n) = number of patients with event). AJCC stage is dichotomized in: stages I-III (disease specific survival at 10 years >60%) / stage IV (Disease specific survival at 10 years <50%) and TERT expression status in: positive / negative. Forward stepwise (Likelihood Ratio) regression method was used for multivariate modelling on PFS and overal survival. HR: hazard ratio; 95% CI: confidence intervals. **C.** Receiver-operating characteristic (ROC) curve analysis of individual variables and their combinations for the prediction of progressive disease. AUC: area under the curve, 95% CI: Confidence Intervals.

Figure S3

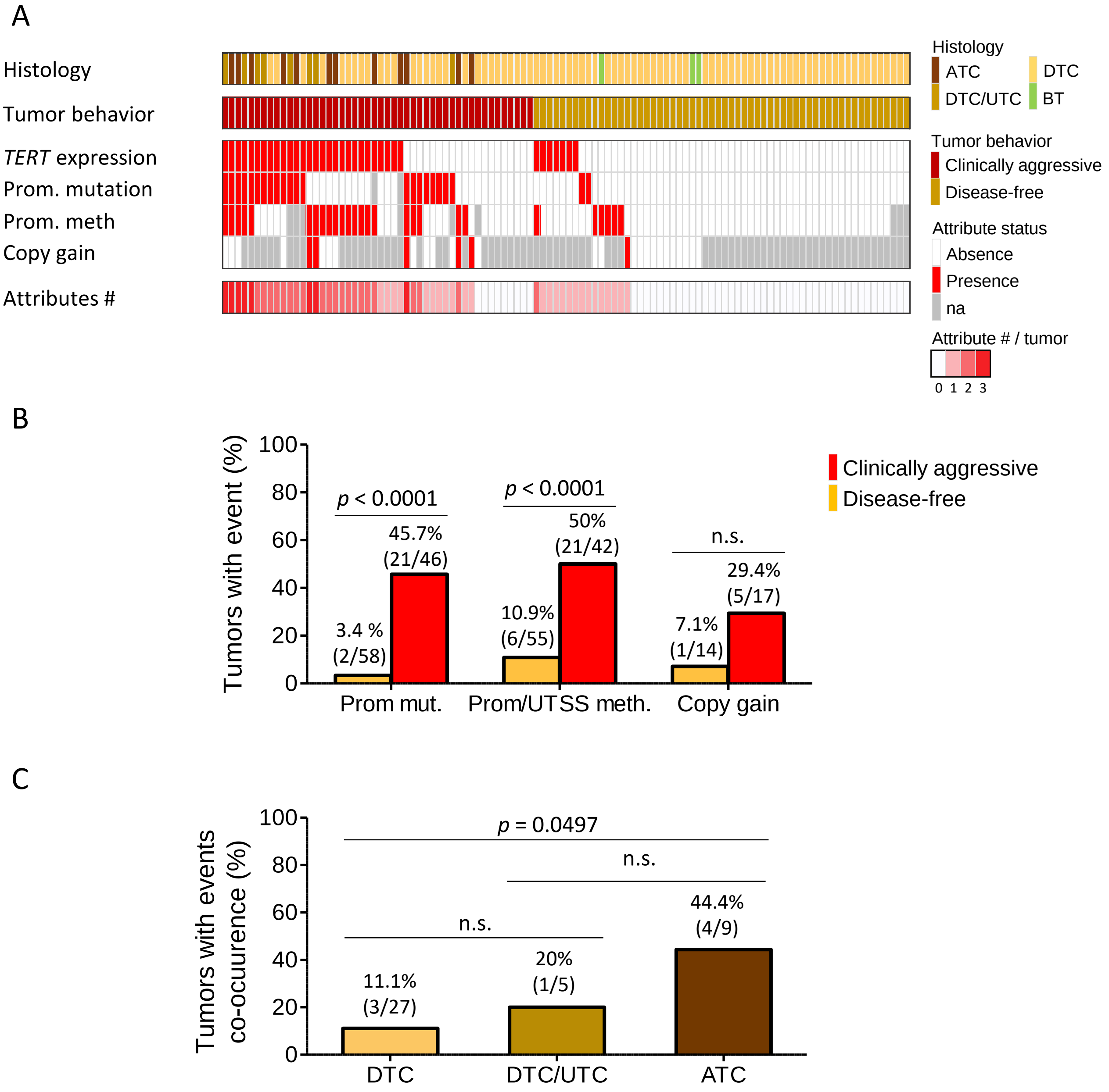

Figure S3. **Poor prognosis associated events in TERT are significantly more frequent in clinically aggressive thyroid tumors than in primary tumors from disease-free patients. A** Print of *TERT* events for the analyzed tumor series. Columns represent tumors and rows TERT events. *TERT* expression (n = 106), promoter mutation (n = 104), promoter methylation (> 16.1%) (n = 97) and copy number data of *TERT-locus* for a subset of tumors (n = 31) are ranked according to the clinical behavior of the disease. ATC: Anaplastic Thyroid Carcinomas; DTC: Differentiated TC; DTC/UTC: Differentiated TC with an undifferentiated (poorly differentiated/anaplastic) component; BT: Benign Tumor. Attribute # indicates the number of events affecting *TERT* per tumor. **B.** Interleaved bar graph representing the percentage of clinically aggressive and disease-free tumors with indicated *TERT* events. Two-sided Fisher exact test p-values are represented. **C.** Percentage of clinically aggressive tumors with co-occurrence of *TERT* promoter hypermethylation and mutation (events) according to histological classes. ATC: Anaplastic Thyroid Carcinomas; DTC: Differentiated TC; DTC/UTC: Differentiated TC with an undifferentiated (poorly differentiated/anaplastic) component. One-sided Fisher exact test *p*-value are shown. n.s.: not statistically significant.

Figure S4

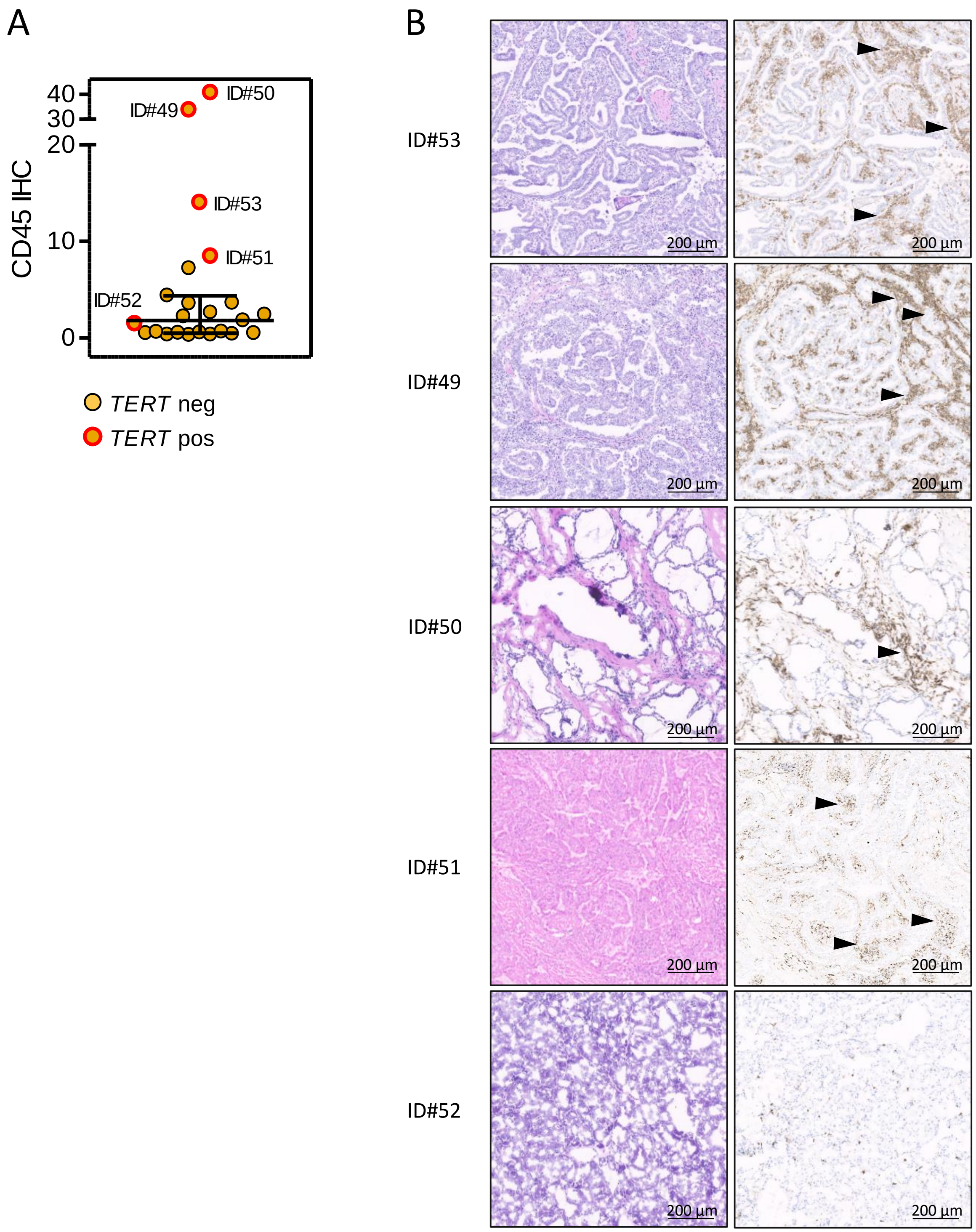

Figure S4. Lymphocytic infiltration and *TERT* expression in thyroid tumors with excellent response to primary treatment. **A.** CD45 (leukocyte common antigen, LCA) immunostaining plot for automatic quantification of complete sections of differentiated thyroid tumors with excellent response to primary treatment (n=23) using the ZEISS ZEN 3.1 software. Dots represent CD45 positive area (x100) relative to total area. Median and interquartile range are shown. Red border dots indicate *TERT*-expressing tumors (*TERT* pos); Black border dots indicate tumors with negative *TERT* expression (TERT neg). All specimens were frozen-preserved. **B.** Representative images of H&E staining (left panel) and CD45 (leukocyte common antigen, LCA) immunostaining (right panel) of the *TERT*-expressing thyroid tumors with excellent response to primary treatment. Staining of all specimens with available material (5/6) is shown. Black arrows point to lymphocytic infiltrates. Only ID#52 was negative for this trait.

Figure S5

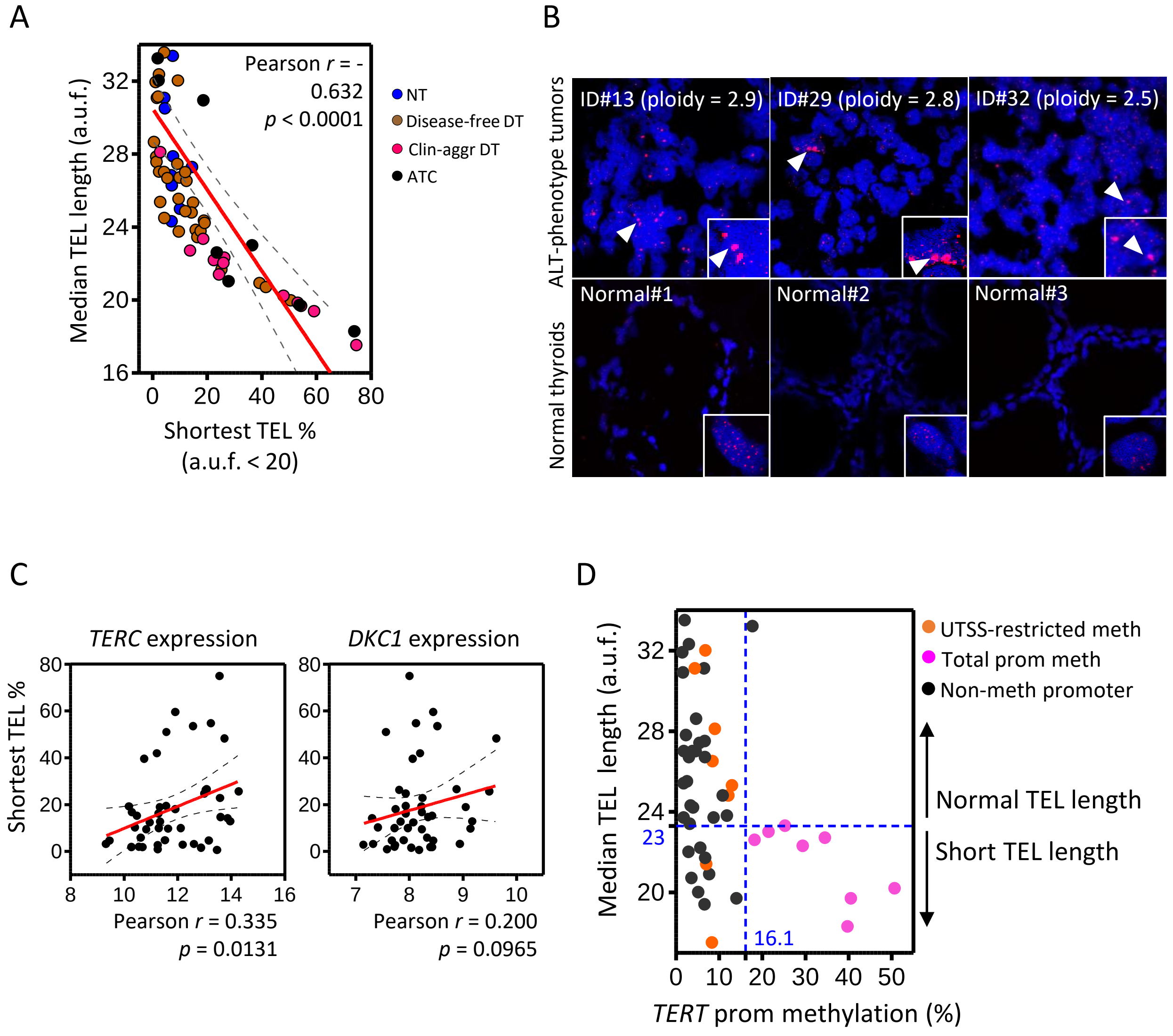

Figure S5. Tumor telomere (TEL) length and immortalization hallmarks. A. Linear regression plot for shortest telomere percentage and median telomere (TEL) length (a.u.f.: arbitrary units of fluorescence). Each dot represents a thyroid tissue. NT: normal thyroids, DT: differentiated tumors; ATC: anaplastic thyroid carcinomas. Pearson *r* coefficient and two-tailed *p*-value are shown. Bright blue dashed line represents short telomere median length cut-off. **B.** Representative telomere-specific FISH images for anaplastic thyroid carcinomas (ATC) showing ALT-associated telomeric *foci* (upper panel, white arrows) and for 3 normal thyroid tissues (lower panel) showing regular telomeric signals. Image magnifications are included in the lower right corner of each image. Tumor ploidy estimated from SNP array data and the ASCAT bioinformatics approach is included. White arrows point to large, intranuclear, ultrabright FISH signals distinctive of ALT-phenotype. **C.** Linear regression plots of differentiated thyroid tumors for median telomere (TEL) lengths (x-axis) and *TERC* or *DKC1* gene expression (y-axis). Pearson *r* coefficient and one-tailed *p*-value are shown. **D.** XY plot for *TERT* promoter methylation percentage (%) (x-axis) and median telomere (TEL) length (a.u.f.: arbitrary units of fluorescence) (y-axis) of all thyroid tumors with available data for both variables (n=50). Bright blue dashed vertical line indicates the cut-off (≥ 16.1 %) for considering *TERT* promoter methylation; bright blue dashed vertical line indicates the threshold of short telomeres (≤ 23 a.u.f.).

Figure S6

A

	GO:0070034 (GOTERM_MF_DIRECT) Telomerase RNA binding		GO:0005697 (GOTERM_CC_DIRECT) Telomerase holoenzyme complex	
	In term	Not in term	In term	Not in term
Subtelomeric	6	1806	6	1925
Non-subtelomeric	10	15059	15	16278
	p-value = 0.0047		p-value = 0.0188	

B

Chr_arm	SIZE (#genes)	Enrichment Score (ES)	Normalized (ES)	Nominal p-val	False Discovery Rate (FDR) q-val	Familywise-error rate (FWER) p-val
**7_P**	38	0.517	**1.900**	0.000	**0.011**	**0.008**
**5_P**	26	0.540	**1.798**	0.002	**0.021**	**0.029**
**16_Q**	69	0.411	**1.771**	0.000	**0.021**	**0.044**
**16_P**	189	0.322	**1.658**	0.000	**0.043**	0.124
22_Q	52	0.397	1.606	0.010	0.054	0.183
X_P	27	0.464	1.589	0.020	0.051	0.207
8_Q	73	0.329	1.413	0.028	0.166	0.584
5_Q	76	0.314	1.366	0.034	0.191	0.700
18_P	24	0.400	1.324	0.096	0.219	0.783
19_P	160	0.236	1.184	0.105	0.438	0.966
9_Q	106	0.245	1.141	0.180	0.489	0.983
X_Q	83	0.257	1.129	0.228	0.475	0.985
17_P	95	0.238	1.086	0.269	0.542	0.998
1_P	82	0.245	1.079	0.272	0.521	0.998
14_Q	58	0.254	1.061	0.324	0.528	0.998
7_Q	22	0.330	1.054	0.355	0.510	0.999
21_Q	75	0.226	0.986	0.483	0.638	0.999
20_P	76	0.226	0.977	0.488	0.625	1.000
19_Q	170	0.183	0.911	0.703	0.756	1.000
20_Q	77	0.181	0.792	0.882	0.964	1.000
3_Q	63	0.192	0.791	0.884	0.921	1.000
1_Q	40	0.198	0.759	0.896	0.924	1.000
10_P	21	0.169	0.552	0.975	0.993	1.000
4_P	69	-0.177	-0.716	0.953	0.913	1.000
15_Q	26	-0.223	-0.727	0.863	0.954	1.000
17_Q	90	-0.178	-0.741	0.934	0.998	1.000
18_Q	17	-0.280	-0.809	0.740	0.954	1.000
6_P	31	-0.276	-0.913	0.592	0.786	1.000
13_Q	47	-0.249	-0.924	0.563	0.820	1.000
12_Q	31	-0.274	-0.938	0.567	0.849	1.000
10_Q	35	-0.288	-0.992	0.487	0.768	1.000
2_P	18	-0.337	-1.000	0.460	0.818	1.000
11_P	102	-0.239	-1.010	0.435	0.877	1.000
2_Q	52	-0.279	-1.065	0.334	0.786	1.000
11_Q	17	-0.441	-1.288	0.145	0.293	0.944
6_Q	30	-0.402	-1.350	0.103	0.235	0.865
8_P	13	-0.515	-1.385	0.103	0.221	0.778
12_P	39	-0.394	-1.421	0.037	0.222	0.694
9_P	22	-0.469	-1.470	0.054	0.214	0.575
4_Q	16	-0.516	-1.482	0.060	0.296	0.545
3_P	12	-0.565	-1.503	0.062	0.513	0.494

C

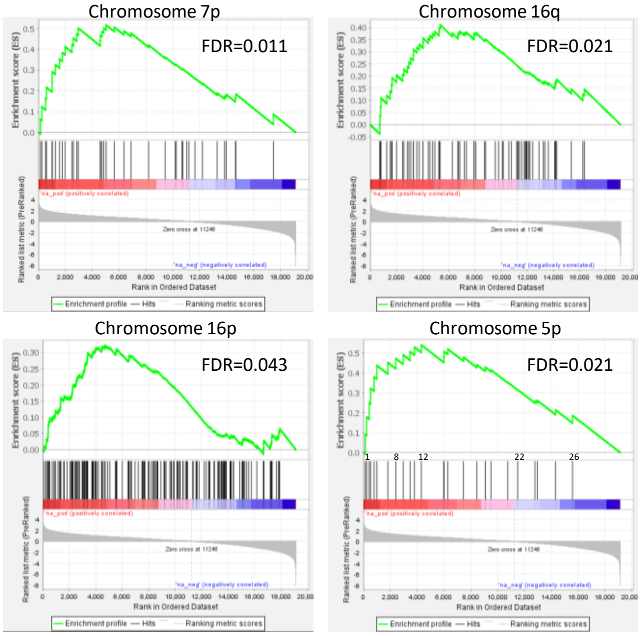

1. *LPCAT1* 2. *SLC12A7* 3. *EXOC3* 4. *BRD9* 5. *SDHA* 6. *NKD2* 7. *IRX2* 8. *TERT* 9. *SDHAP3* 10. *CCDC127* 11. *IRX1* 12. *C5orf38* 13. *TRIP13* 14. *IRX4* 15. *CEP72* 16. *SLC6A3* 17. *NDUFS6* 18. *AHRR* 19. *MIR4635* 20. *ZDHHC11* 21. *LRRC14B* 22. *SLC9A3* 23. *MRPL36* 24. *CLPTM1L* 25. *PDCD6* 26. *TPPP* 27. *PLEKHG4B*

Figure S6. **GSEA results for genes located within the 5 Mb-ends of the chromosomes.** RNAseq genes are ranked based on the limma moderated t-statistic obtained from the gene expression comparison between short telomere length tumors and normal telomere length tumors. **A.** Contingency table of telomerase-related terms significantly enriched in subtelomeric genes (genes within the 5Mb end of chromosomes). P-value of Fisher's exact test for each term are shown. Subtelomeric genes in both terms are *TERT*, *DKC1*, *NHP2*, *HNRNPU*, *SNRPB* and *SMG6*. These results are subtracted from the DAVID functional annotation enrichment analysis of subtelomeric genes using the Gene Ontology (GO) databases: GOTERM_BP_DIRECT, GOTERM_CC_DIRECT and GOTERM_MF_DIRECT. Analyses are adjusted for the number of genes in the lists (subtelomeric / non-subtelomeric) with annotation in each of the databases. **B.** All chromosome ends (5 Mb ends) table with the enrichment analysis statistics for high expressed genes in short telomere tumors (n = 10) compared with normal telomere tumors (n = 5). In bold are highlighted the regions with the more significant enrichments (FDR p-val < 0.05). Significant FDR and FWER p-val are highlighted in red. **C.** GSEA enrichment plots of the chromosome ends (5 Mb ends) with acute significant enrichments (FDR q-val < 0.05) for upregulated genes in short telomere length tumors compared to normal telomere length ones. Gene showing higher expression levels either for short telomere or normal telomere tumors are located in the edges of the bar. The black lines represent the subtelomeric genes (hits) location in the whole ranked list (including subtelomeric and non-subtelomeric genes). The green line and the grey distribution represent the enrichment score and the ranking metric score, respectively. The distribution of these genes was found biased to short telomere length tumors for the represented chromosome 5 Mb ends. Gene list of chromosome 5p end is included and ordered according to their position in the corresponding GSEA plot ranking metric score. In red are highlighted those genes with a limma t-statistc ≥ 1.5.

Figure S7

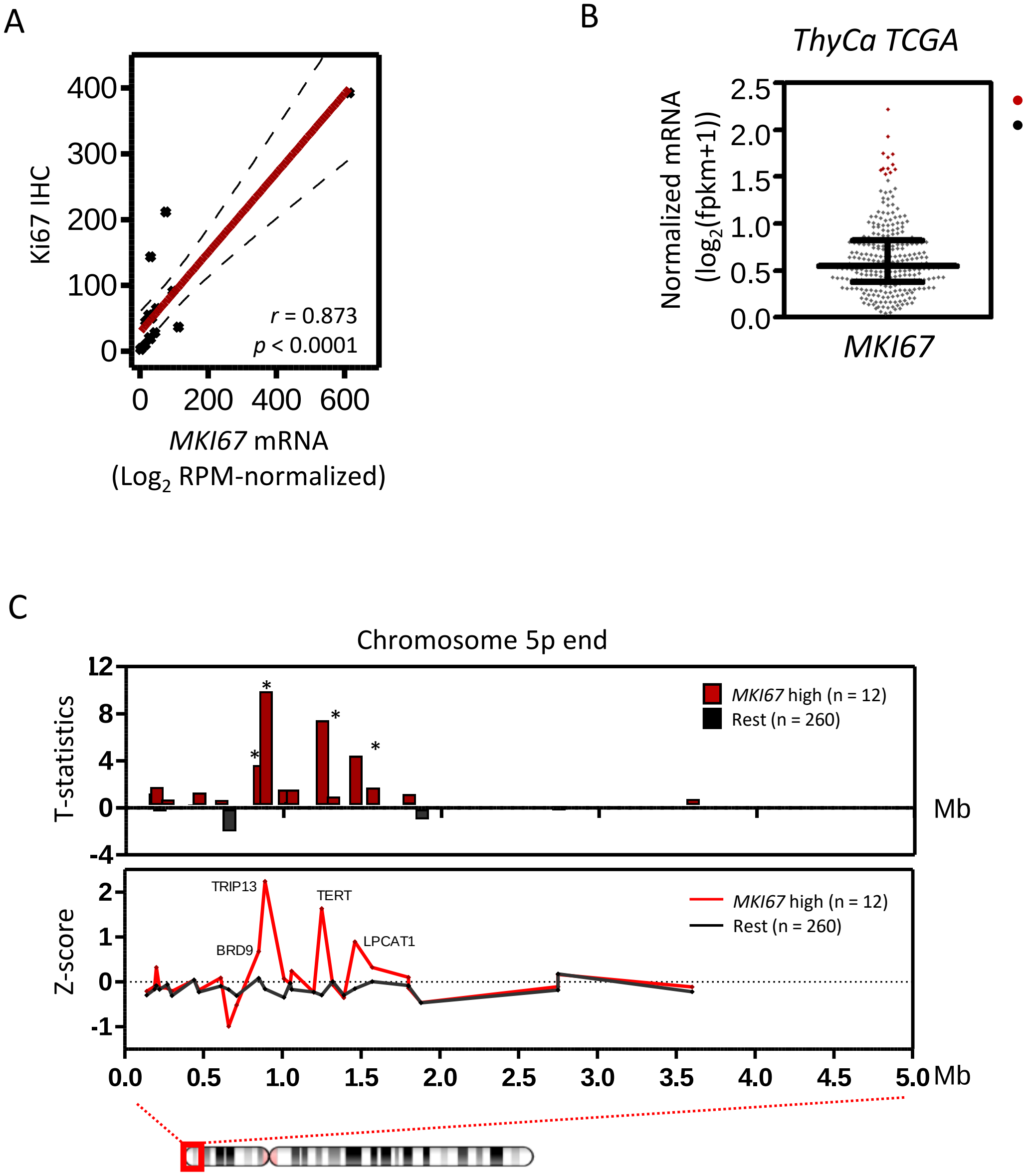

Figure S7. **TCGA series high expressors of *MKI67* gene showed an upregulation of chromosome 5p-end genes. A.** Linear regression plot for *MKI67* mRNA and protein abundancy of the tumors included in Figure 4. mRNA and protein data were obtained from RNA sequencing analysis and ZEISS ZEN 3.1 software automatic analysis of Ki-67 immunohistochemical staining, respectively. Dashed line curves indicate 95% confidence intrevals. **B.** *MKI67* gene expression in TCGA tumor series (n = 272). Gene expression median value and interquartile ranges are represented. In red are highlighted TCGA tumors with outlier high expression of *MKI67* (> Q3 + (IQR*1.5)). **C.** Plots showing moderated T-statistics from limma t-test comparison between *MKI67* high TCGA tumors and the rest of tumors (upper panel) and gene expression z-score (down panel) of 5 Mb subtelomeric genes of chromosome 5p. Dark red bars indicate genes upregulated in *MKI67* high TCGA tumors; grey bars represents genes upregulated in the rest of TCGA tumors. The bar position reflects the gene location on the chromosome. The height of the bars is proportional to the T-statistics of expression between *MKI67* high expressors and the rest of TCGA tumor. Only TCGA tumors with a purity higher than 60% were considered (n=272). Asterisk denote FDR p-value ≤ 0.05. TCGA RNAseq (log2(FPKM+1)) and clinicopathological data were downloaded from the UCSC Xena web-tool (http://xena.ucsc.edu; TCGA Thyroid Cancer (THCA) study).

Figure S8

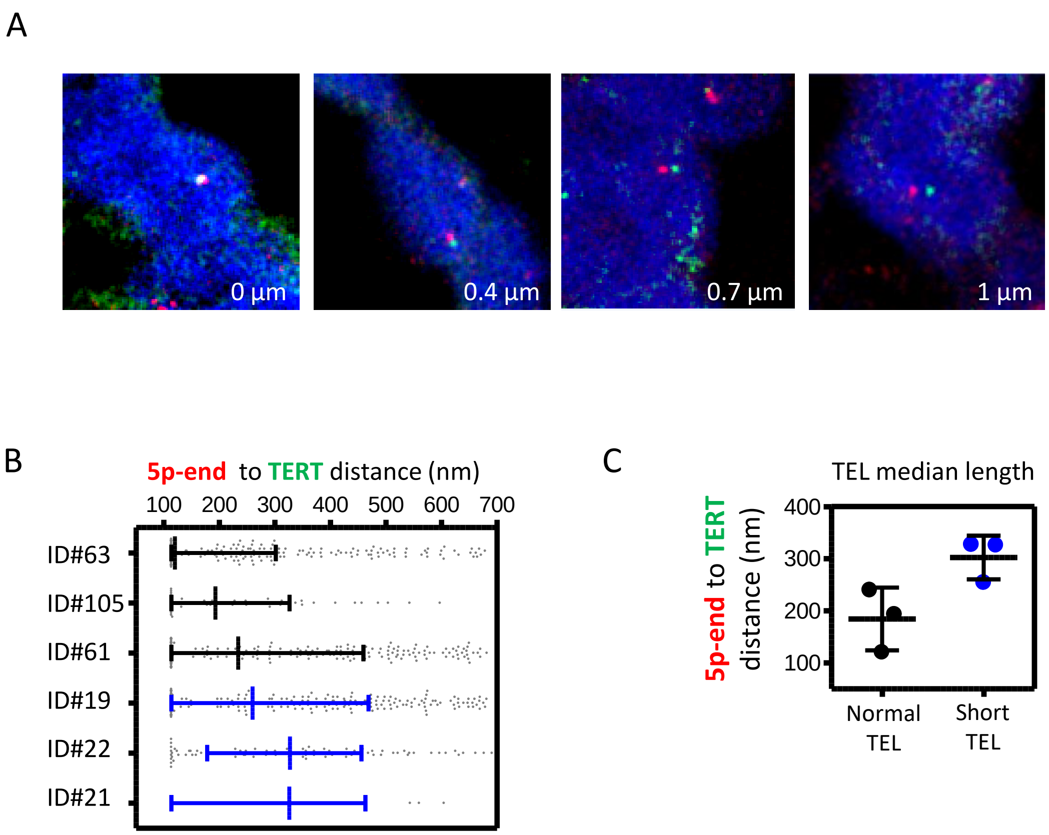

Figure S8. High-resolution confocal microscope analysis of 5p-end and *TERT* signal pair distances for a set of thyroid tumors. A. Thyroid tumor images captured with SP8 high-resolution confocal microscope showing different distances between 5p-end and *TERT* signals. A maximum red-green signal distance of 0.7 µm (6 pxl) was consider as signal pair. **B.** Scatter plot of the distances (nm) between 5p-end signal and the nearest TERT signal for 6 thyroid tumors measured by high-resolution confocal microscopy. **C.** Scatter plot of the median distances (nm) of 5p-end signal to nearest *TERT* signal for above thyroid tumors gouped according to their telomere median length (TEL). Black dots, tumors with normal telomere lengths (normal TEL, n = 3); bright blue dots, tumors with significant telomere shortening (short TEL, n = 3). Mean values are plotted with SD for each tumor group. Two-tailed unpaired t-test p-value = 0.0494.

Figure S9

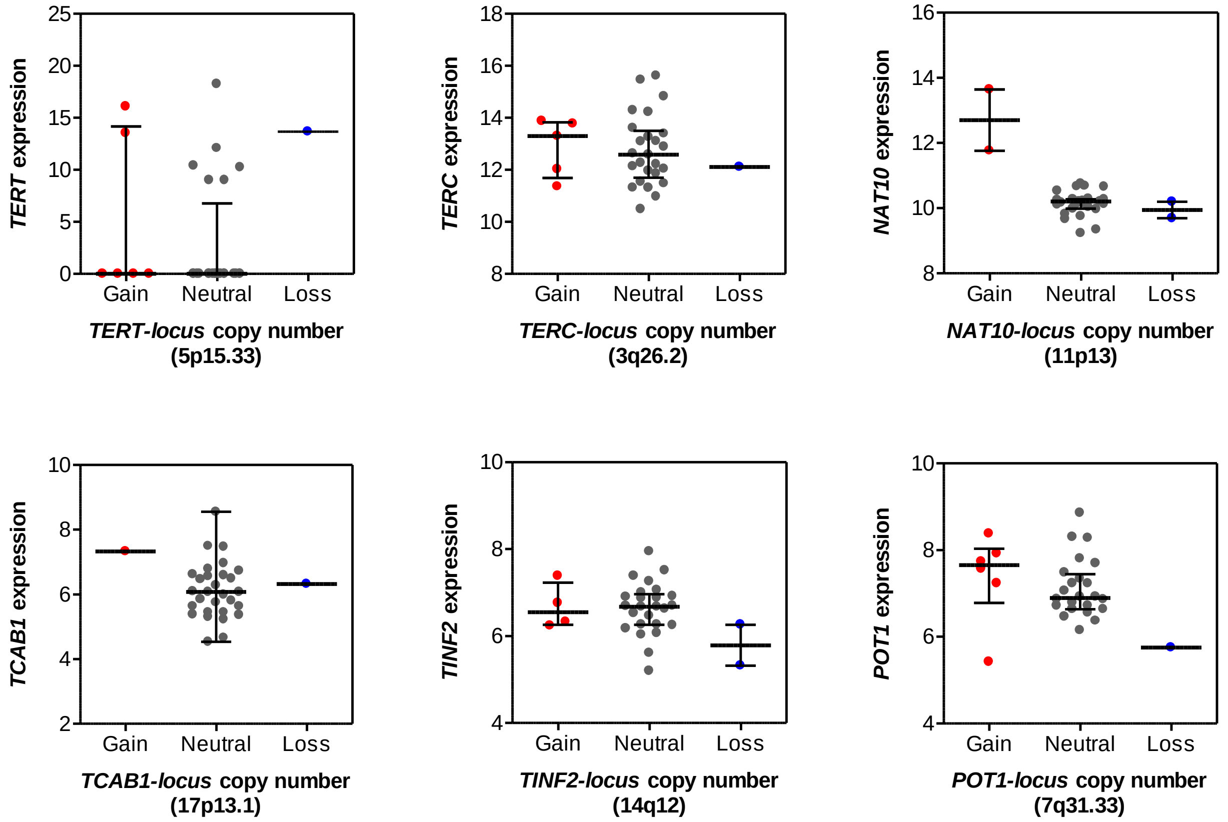

Figure S9. Scatter plots representing normalized gene expression of a subset of 31 tumors from the original series divided according to gene *locus* copy number *status*. Gain: copy number of indicated gene *locus* > 2; Neutral: copy number of indicated gene *locus* = 2; Loss: copy number of indicated gene *locus* < 2. Gene expression median and interquartile range values are represented for each copy number class. Only autosomal chromosomes were included in copy number analysis.

Methods Table S2. Sequences of the primers for the identification of *TERT* promoter mutations (*TERT* prom mut) and methylation levels (THOR_A1-A4).

Amplicon	Primer	Sequence (Adaptor + Primer) 5'-3'
***TERT* prom mut**	Forward	TCGTCGGCAGCGTCAGATGTGTATAAGAGACAGCAGCGCTGCCTGAAACTCG
	Reverse	GTCTCGTGGGCTCGGAGATGTGTATAAGAGACAGGTCCTGCCCCTTCACCTTC
**THOR_A1**	Forward	TCGTCGGCAGCGTCAGATGTGTATAAGAGACAGGGAGGGGTTGGGAGGGTT
	Reverse	GTCTCGTGGGCTCGGAGATGTGTATAAGAGACAGCCTACCCCTTCACCTT
**THOR_A2**	Forward	TCGTCGGCAGCGTCAGATGTGTATAAGAGACAGAGTTGGAAGGTGAAGGGGTAGG
	Reverse	GTCTCGTGGGCTCGGAGATGTGTATAAGAGACAGAACTCCCAATAAATTC
**THOR_A3**	Forward	TCGTCGGCAGCGTCAGATGTGTATAAGAGACAGGAATTTATTGGGAGTT
	Reverse	GTCTCGTGGGCTCGGAGATGTGTATAAGAGACAGTCCCTACACCCTAAAAA
**THOR_A4**	Forward	TCGTCGGCAGCGTCAGATGTGTATAAGAGACAGGTTTAGGTTGTGGGGTAATT
	Reverse	GTCTCGTGGGCTCGGAGATGTGTATAAGAGACAGCTAAAAACAACCCTAAATC
